# When storms slow down: urban effects on rainfall accumulation and flood hazard

**DOI:** 10.1038/s44304-025-00158-4

**Published:** 2025-12-20

**Authors:** Herminia Torelló-Sentelles, Marika Koukoula, Gabriele Villarini, Francesco Marra, Nadav Peleg

**Affiliations:** 1 Institute of Earth Surface Dynamics, University of Lausanne, Lausanne, Switzerland; 2 Department of Civil and Environmental Engineering, Princeton University, Princeton, NJ USA; 3 High Meadows Environmental Institute, Princeton University, Princeton, NJ USA; 4 Department of Geosciences, University of Padova, Padova, Italy; 5 Expertise Center for Climate Extremes, University of Lausanne, Lausanne, Switzerland

**Keywords:** Climate sciences, Atmospheric science

## Abstract

Changes to convective storm motion over urban areas may have important implications on rainfall accumulation and flood risk. Here, we investigate speed changes in storms passing over cities using weather radar data and convection-permitting numerical simulations. The observational analysis consists of tracking individual rain cells across eight cities and comparing movement speeds near the cities relative to a control upwind region. Second, we simulate ten heavy rainfall events crossing Indianapolis, Indiana, and compare cloud-layer horizontal wind speeds from two scenarios: one with and one without the city. We find that the speed of the observed rain cells decreases over and downwind of five urban areas, and seven simulations reveal dampened cloud-layer wind speeds over Indianapolis. Stronger updrafts induce horizontal wind slowing, driven by the warm urban surface. We conclude that rainfall intensification is the primary driver of enhanced urban rainfall accumulation, yet storm slowing contributes to more frequent and stronger enhancements.

## Introduction

With more than half of the world’s population living in cities, and urban expansion and population growth projected to increase^1,2^, exposure to urban floods is becoming increasingly widespread^3^. Flood risk increases in urban areas due to a combination of factors. Changes in land surface decrease surface permeability^4,5^, and short-duration rainfall extremes, which are frequently linked to flash floods, have often been shown to be intensified by both global warming and urbanization^6–10^. Urban rainfall modification tends to result from three main mechanisms: first, changes to the surface energy balance frequently lead to temperature gradients between cities and their surroundings, causing the well-known Urban Heat Island (UHI) effect^11^, which usually enhances convection and intensifies rainfall^12–15^. Second, changes in surface roughness can dynamically change surface winds and convergence patterns near a city, affecting the intensity and spatial patterns of precipitation^16–18^. Lastly, increased atmospheric aerosol concentrations over cities can alter microphysical cloud properties, either enhancing or suppressing rainfall^19–21^.

Aside from modifying heavy rainfall properties, such as its amount, intensity, structure, and occurrence [e.g., refs. ^22–29^], there is also evidence suggesting that urban areas can impact the temporal structure of rainfall, i.e., by altering its timing and duration^30–33^. A suggested mechanism for these observed changes is that a dry urban surface may inhibit the transportation of moisture and slow down the precipitation development process^34^. Another hypothesis is that an urban cluster can slow down or block the movement of precipitation through a building-barrier effect resulting from increased surface roughness^32–35^. Similarly, urban areas have also been found to influence storm movement and dynamics by modifying airflow patterns. For example, convective precipitation can bifurcate around a city^30,35–37^ or its movement can be deviated toward it^38^.

Urban effects on canopy-layer winds, especially near-surface winds, have been extensively studied. Most studies find that near-surface winds are reduced over urban areas, and that this is due to the frictional drag effect from the increased surface roughness of urbanized areas^39–42^. Aside from this dynamic process, the thermodynamics of urban settings have also been shown to impact urban canopy wind speeds. This tends to occur when the background wind speeds are weak, and the UHI effect is strong, resulting in low-level wind acceleration toward and convergence over the thermal disturbance^43–47^. However, airflow modifications above the lower atmospheric layers, where cloud advection mostly occurs, remain less understood. Some evidence suggests that urbanization can influence wind patterns beyond the near-surface. For example, Loose and Bornstein^48^ found that synoptic-scale frontal movement was slowed over an urban area, or across its upwind side in the case of a strong UHI. Few studies have also shown that urban boundary layer wind patterns may be slowed down due to increased atmospheric instability and vertical mixing^47,49^. It is therefore important to further our understanding of urban boundary layer airflow changes because airflow directly influences the movement of precipitation fields.

The changes in the speed of convective storms over urban areas can have important implications on flood risk; for example, the slowing down of a precipitation system may lengthen heavy rainfall duration, and lead to increases in accumulated rainfall and pluvial-flood risk^50–56^. Changes in the speed of future mesoscale convective systems as a result of global warming are foreseen, with regional variations^9,50^. Namely, in Europe, heavy rainstorms that are more intense have a reduced storm speed and longer duration, and are predicted to become more frequent under climate change^57^. Here, we hypothesize that urban areas may affect heavy precipitation motion due to thermodynamic and dynamic processes that result from the modified land surface.

We aim to determine whether and how the speed of convective storms is altered over cities. We investigate how urban areas affect the speed of heavy rainfall systems by using: (i) 7 years of weather radar observations (2015–2021), and (ii) high space-time resolution simulations from a convection-permitting model. First, we identify heavy rainfall events that passed near eight urban areas located in different climates and characterized by varying forms, and track their trajectories with a storm-tracking algorithm. We then compare changes in the distributions of rain cell speeds upwind, over, and downwind of each city, relative to an upwind control area that is further away from and assumed to be unaffected by the city. Second, we focus on one study area, the city of Indianapolis (Indiana, U.S.), and evaluate changes in horizontal wind speeds over the urban area where the rain-producing clouds are embedded. We do this by comparing an “urban” simulation (with the land cover of Indianapolis) to a “no-urban” simulation (where the area of Indianapolis is replaced with the most common surrounding land cover) for 10 rainfall events. Third, we explore mechanisms that are likely responsible for the observed storm motion changes. Finally, we assess the contribution of these speed changes to rainfall accumulation over the city. Our findings reveal decreases in storm speed across multiple urban areas and their potential to enhance rainfall accumulation over cities.

## Results

### Rural–urban rain cells’ speed changes from radar observations

**Fig. 1:**
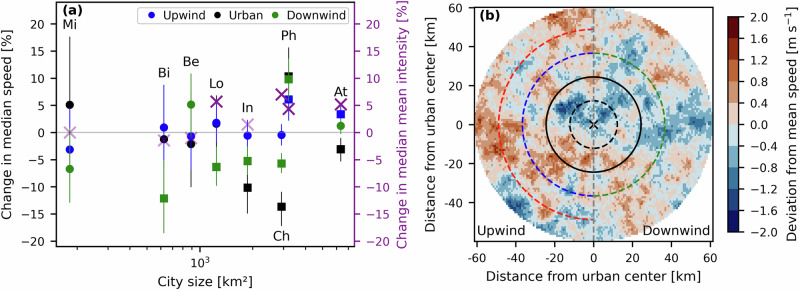
Changes in rain cell speed between urban areas and surrounding regions derived from weather radar data. **a** Change in median object speed from the control to the three urban bins, plotted against city size. The color of the points refers to the urban bin, and the error bars represent the 95% confidence intervals of the median differences. Square (round) symbols refer to (not) significant change (*p* < 0.05). Purple crosses mark the median change in object mean rainfall intensity from the control to the central urban bin. Opaque crosses indicate statistically significant changes, while transparent crosses indicate non-significant changes. Cities are labeled by their two-letter acronyms: Mi (Milan), Bi (Birmingham), Be (Berlin), Lo (London), In (Indianapolis), Ch (Charlotte), Ph (Phoenix), and At (Atlanta). **b** Composite of mean rainfall object speed over Indianapolis, expressed as the deviation from the mean. Each rainfall object is rotated around a fixed center located at Indianapolis’s city center, based on the corresponding track’s mean direction of motion. For all tracks, the downwind direction is to the east, and the upwind direction is to the west. The black circle indicates the average radius of the city, and the city center is marked with a cross. The black, blue, green, and red dashed lines indicate the edges of the urban, upwind, downwind, and control bins, respectively.

The changes in median rain cell speed and mean rainfall intensity from a control region located at the city’s surroundings (assumed to be unaffected by the urban area) to three urban locations (upwind, over, and downwind of the urban center) were quantified for the study cities (see land cover and urban structure maps in Fig. S1 and illustration of bins in Fig. S2). Figure 1a reveals that five out of the eight cities (Birmingham, London, Indianapolis, Charlotte, and Atlanta) showed statistically significant negative changes in rain cell speed at least over one urban location; usually on the downwind side and/or over the cities. The median rain cell speeds decreased by 12.2, 6.4, 10.2, 13.7, and 3.1%, respectively. In addition, statistically significant increases in rain cell mean rainfall intensity occurred for the five cities, either over the urban area or on its downwind side (Figs. 1a and S3), with median increases in mean intensity ranging from 4 to 7%. Generally, the largest cities showed the greatest and most statistically significant changes in both rain cell properties.

The composite map of mean rain cell speed differences over Indianapolis is presented in Fig. 1b. Rainfall objects were rotated so that rainfall motion is from west to east. We observe that speed decreases occurred generally over the urban core and extended further downwind of it up to 60 km. The largest magnitude of these speed decreases was approximately 2 m s^−1^. Composite maps of the speed differences for the other study cities are provided in Figs. S4 and S5. As in Fig. 1a, Birmingham, London, Charlotte, and Atlanta displayed slower rainfall motion either above their centers, downwind of them, or both. In Birmingham, speed decreases were observed over the urban core and on the downwind side of the city, extending up to 20 km from its center. In Charlotte, the strongest decreases occurred within the city, particularly on the downwind side, reaching up to 35 km from the center. In London and Atlanta, decreases were concentrated downwind of the urban centers, extending up to 40 km and 60 km, respectively. In both cities, the upwind region (up to 40 km) showed increases in rain cell speeds (Figs. 1a, S4, and S5). We note that the speed anomalies were fairly heterogeneous across the studied domains, showing considerable spatial variability. Nevertheless, the consistent pattern of negative anomalies over the five cities suggests a consistent slowing-down urban signal, in addition to the statistically significant changes reported in Fig. 1a.

### Rural-urban changes in cloud-layer wind speeds from numerical simulations

**Fig. 2:**
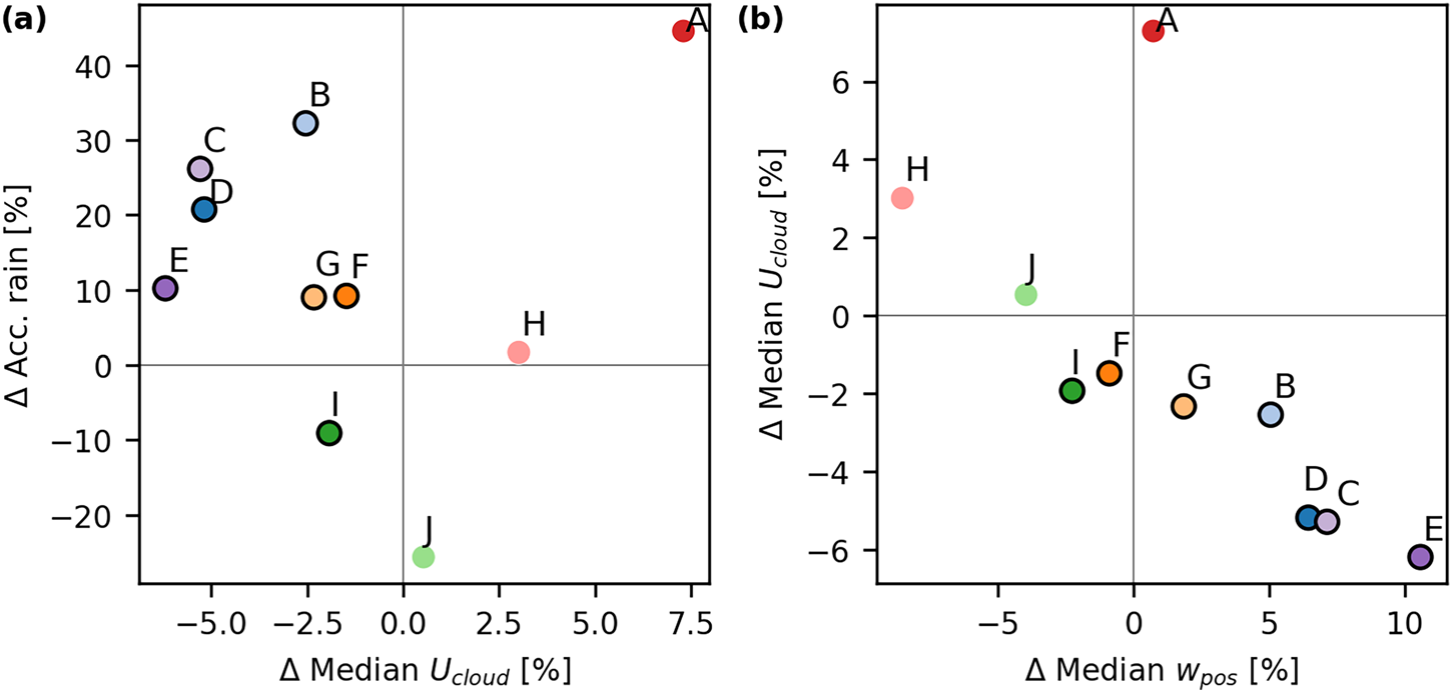
Urban impacts on accumulated rainfall, cloud-layer wind speeds (*U*_*cloud*_), and uplift (*w*_*pos*_) from WRF simulations. Scatter plots of (**a**) the difference in accumulated rainfall (Δ*Acc*. *rain*) plotted against the median difference in *U*_*cloud*_, and **b** the median difference in *U*_*cloud*_ versus the difference in median *w*_*pos*_. All differences are between the “urban” and “no-urban” simulations and are expressed as percentages relative to the “no-urban” case. The events that showed decreases in *U*_*cloud*_ are outlined in black.

After exploring climatological rainfall speed changes across multiple cities using weather radar data, we focus on Indianapolis and explore the driving processes affecting urban-induced rainfall speed changes by simulating several convective rainfall events with an atmospheric numerical model. Median cloud-layer horizontal wind speeds (*U*_*cloud*_) over the urban area were extracted from the convection-permitting numerical simulation outputs for Indianapolis and compared between the “urban” and “no-urban” simulations (section “Methods”). The “urban” to “no-urban” differences were quantified as the “urban” minus “no-urban” case. The model domain setup and land use/land cover maps are shown in Fig. S6. Figure 2a displays that, out of the 10 simulated rainfall events, seven showed reduced *U*_*cloud*_ values over the urban area compared to the “no-urban” case, and the remaining three displayed *U*_*cloud*_ increases. *U*_*cloud*_ decreases ranged from 1.5 to 6.2% and increases from 0.5 to 7.3%. *U*_*cloud*_ decreases occurred along with an increase in accumulated rainfall (enhancement of 9–32%) in the “urban” case for six events (B, C, D, E, F, and G). Conversely, increases in *U*_*cloud*_ were also sometimes accompanied by more accumulated rainfall (events A and H) in the “urban” case. We did not find a relationship between differences in *U*_*cloud*_ and total rainfall accumulation between the “urban” and “no-urban” simulations (Fig. 2a).

We next explored potential mechanisms that explain the *U*_*cloud*_ differences. We found a significant negative linear relationship between the change in *U*_*cloud*_ and positive vertical wind speeds (*w*_*pos*_) over the urban area (*p* = 0.029, *R*^2^ = −0.69; Fig. 2b). We particularly note that the five events showing increased accumulated rainfall and the largest decreases in *U*_*cloud*_ over the city, all exhibited positive increases in *w*_*pos*_ (B, C, D, E, and G). Potential links between *U*_*cloud*_ differences and other convection-related variables were also explored, such as surface and boundary layer UHI, background wind speed, and peak rainfall time; however, no clear relationships to *U*_*cloud*_ were found. A general trend was observed in which events with longer residence times showed stronger slowing effects, although the correlation was weak (Table S1).

**Fig. 3:**
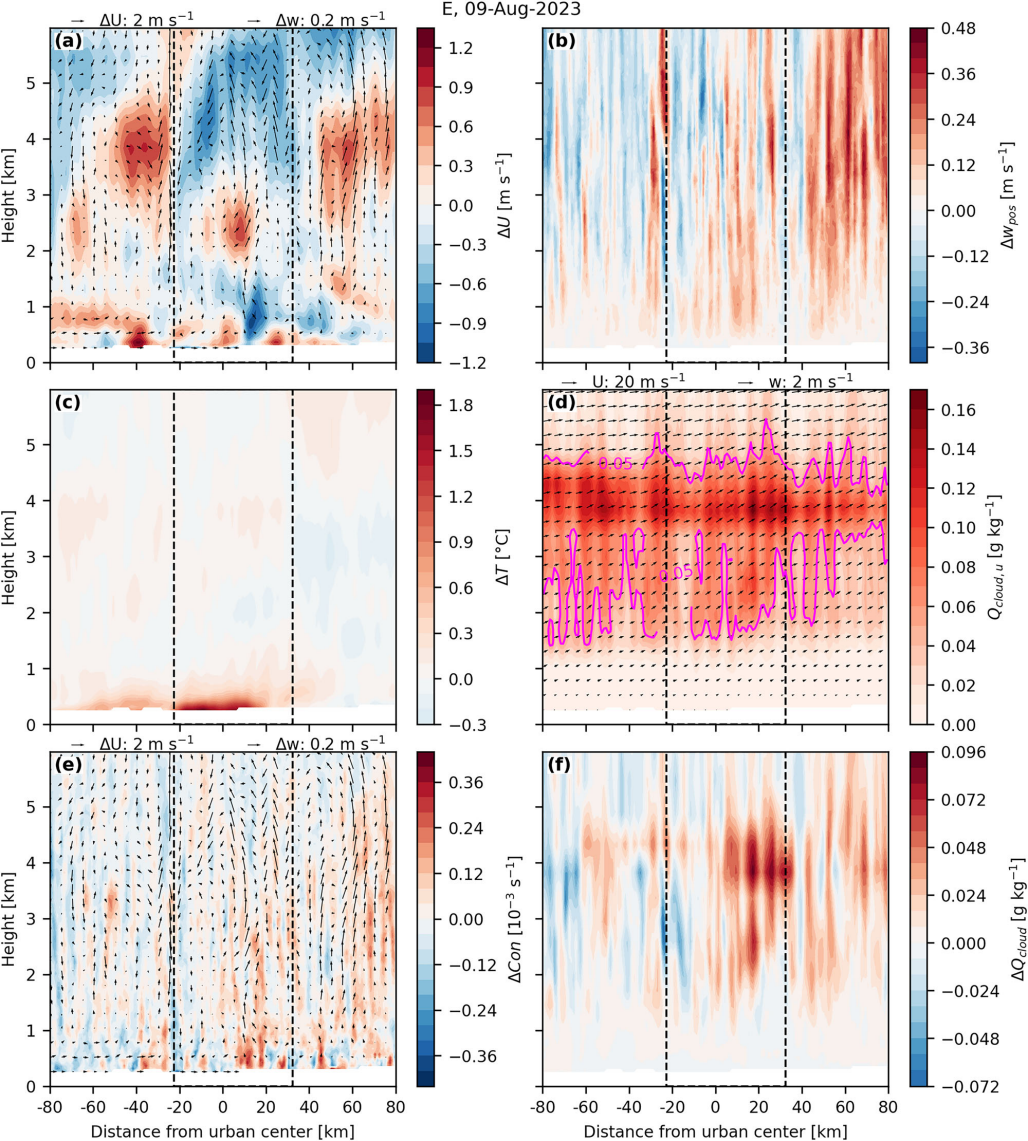
Area-averaged vertical cross-sections of several convection-related variables between the “urban” and “no-urban” simulations of Event E. Changes are presented for (**a**) *U*, **b** *w*_*pos*_, **c** *T*, **e** *Con*, and (**f**) *Q*_*cloud*_. **d** Cloud water mixing ratio (*Q*_*cloud*,*u*_) from the “urban” simulation, with magenta contours indicating values of 0.05 g kg^−1^. In (**d**), vector arrows represent the wind field, with the vertical component indicating vertical motion and the horizontal component representing wind speeds along the cross-section line. In (**a**, **e**), vector arrows show the difference in the wind field between the “urban” and “no-urban” simulations. Note that vertical vectors are scaled by a factor of 10 relative to the horizontal direction. The dashed black lines indicate the extent of the urban area. Distances from the urban center are negative when upwind and positive when downwind.

Figure 3 provides an example of a vertical cross-section for a rainfall event (E) where rainfall accumulation increased, and rainfall advection speed was reduced by the urban area. This example represents the general processes seen across the other slowing events. The cross-section was taken following the mean direction of the winds at 500 hPa and averaged across an area extending 5 km from the city center (see position in Fig. S7). During this event, high values of cloud water mixing ratio (*Q*_*cloud*_), indicating the presence of clouds, were observed between an altitude of 2–5 km (Fig. 3d). Figure 3c shows that there was a moderate UHI (mean temperature difference at 2-m was 1 °C) that developed over the urban area, particularly on its upwind side, and that extended downwind and up to a height of 1 km. We also observed low-level air convergence (*Con*) close to the urban center and low-level airflow directed toward it (Fig. 3a, e). Between the center and the downwind side of the city, there was an area of strong horizontal wind (*U*) reduction and increased *w*_*pos*_ near the surface (Fig. 3a, b). This upward motion extended vertically until the cloud layer (2–5 km), especially on the downwind side of the city, which also displayed clear *U* decreases between 3 and 5 km (Fig. 3a, b). *Q*_*cloud*_ also increased above and downwind of the city (Fig. 3f).

As for the other simulations that showed reduced cloud-layer winds, a UHI effect was also visible between the “urban” and “no-urban” simulations (events B, C, D, F, G, and I; Figs. S8–S13). In some cases (e.g., in C, D, and I), there was also low-level air convergence and enhanced uplift over the urban area, starting at low levels and extending upward to higher atmospheric layers containing the clouds. The hotspots of horizontal wind decrease coincided with areas of increased uplift. In simulations B, F, and G, the urban area did not show strong low-level convergence over the city compared to the other slowing events (Figs. S8, S11, and S12). Increases in vertical motion were also not as strong near the surface and occurred most strongly in higher levels of the atmosphere, around 3–5 km. In these simulations, *U* often decreased in areas experiencing the greatest increases in uplift strength. This indicates that, although the warm surface initiated atmospheric instability and some upward motion, uplift at the cloud layer was enhanced by another process. Figures S8c, S11c, and S12c display warmer cloud temperatures above the city, showing that convective lifting was strengthened due to the warmer and more buoyant air. We note that simulations B, F, and G showed weaker slowing effects compared to the other rainfall events (Fig. 2), indicating that the cloud’s decelerating effect was generally the strongest when there was strong low-level convergence and strong uplift over the city.

The negative relationship between Δ*U*_*cloud*_ and Δ*w*_*pos*_ (Fig. 2b) is also apparent across the different vertical cross-sections (Figs. 3 and S8–S13). When comparing the cross-sections, areas where *U* decreased generally corresponded to areas where *w*_*pos*_ increased. Notably, during the slowing events F and I, the median change in *w*_*pos*_ over the city was small and negative (Fig. 2b). However, both cases exhibited strong localized increases in *w*_*pos*_ within pockets of the urban area, which also coincided with locations of *U* decreases (Figs. S11 and S13).

Two events, H and J, showed increases in *U*_*cloud*_ over the city. These two events showed decreases in uplift strength over the urban area, compared to the other events (Figs. 3b, S14 and S15). This indicates that the same mechanism as for the other events drove the wind speed changes, but in the other direction; weaker uplift over the urban area, compared to the “no-urban” simulation, resulted in faster-moving clouds. During event J, rainfall and convection were suppressed by the urban area, shown by the weaker uplift and decreased rainfall accumulation over the city (Figs. 2 and S7). During event H, although convection was weaker directly over the city, it was enhanced downwind of the city, as shown by the increases in vertical motion (Fig. S14b) and increased rainfall accumulation in that region (Fig. S7). This downwind region also displayed enhanced convergence at the cloud layer (Fig. S14e). Therefore, the increases in *U*_*cloud*_ over the city most likely occurred due to the downwind convergence zone, which caused air to accelerate toward it. This downwind area of enhanced uplift also correlated with an area of *U* decrease, indicating that the slowing of rainfall advection occurred downwind (Fig. S14a, b).

Event A displayed markedly larger changes in *U*_*cloud*_ with negligible changes in uplift over the city compared with the other events, and it does not follow the *U*_*cloud*_-*w*_*pos*_ relationship observed for the others (Fig. 2b). The cross-sections in Fig. S16 reveal that there was a strong convergence zone downwind of the city at an altitude of 1–4 km (Fig. S16e), which was likely caused by the increase in air temperature in that location due to the UHI signal being transported downwind of the urban area (Fig. S16c). This strong area of convergence caused air to accelerate toward it, consequently accelerating cloud-layer wind speeds over the city. In this simulation, two convective systems merged over the city, and the faster advection despite stronger uplift may reflect complex interactions between urban advection and convective-system merging.

### Link between rainfall advection speed and accumulated rainfall from numerical simulations

**Fig. 4:**
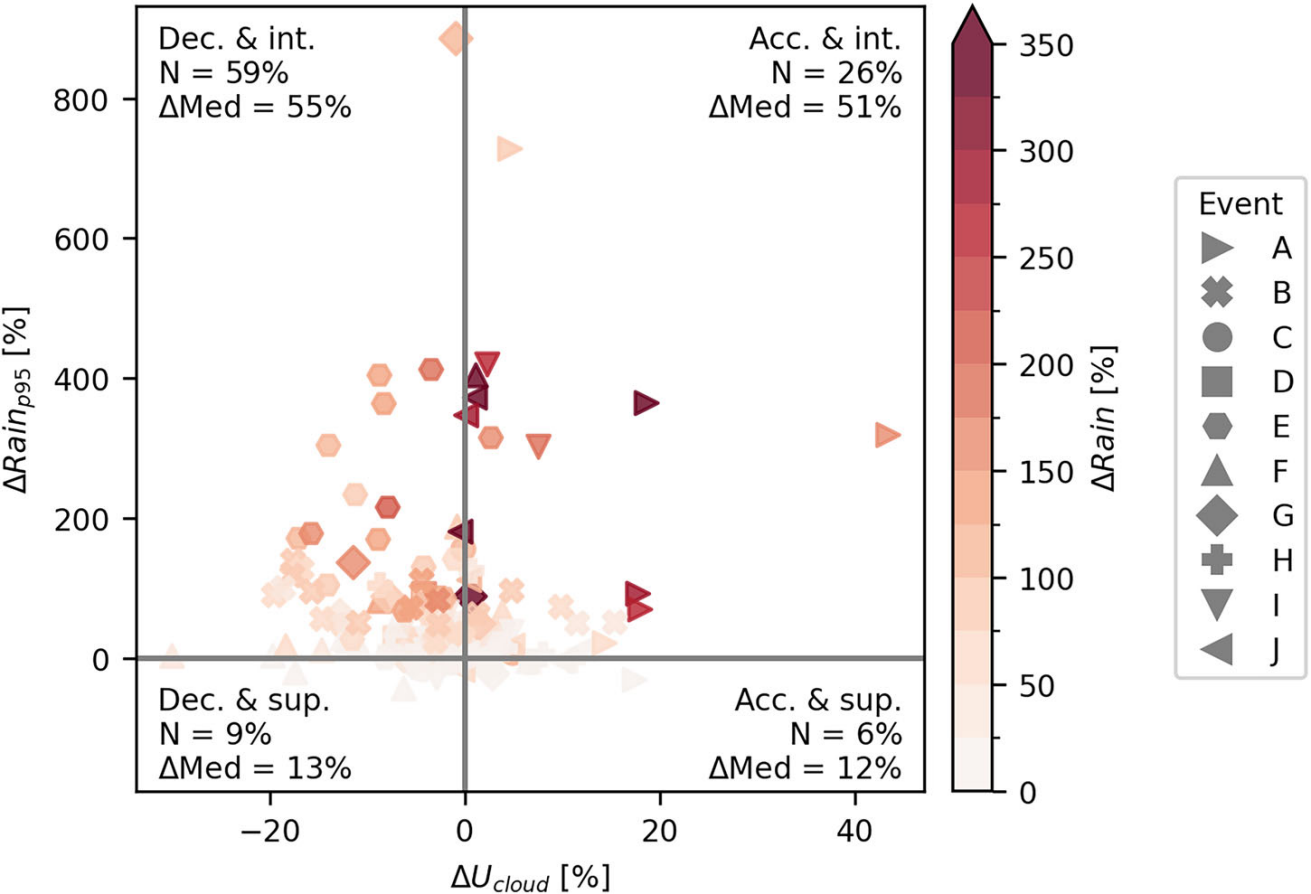
Scatter plot of the change in 95^th^ percentile of accumulated rainfall (ΔRain_*p*95_) versus the change in mean cloud-layer horizontal wind speed (Δ*U*_*cloud*_) over the urban area. The color of the points indicates the change in the mean accumulated rainfall (ΔRain). All changes are computed as percent differences between the “urban” and “no-urban” simulations at each 15-min output time. The symbol’s shape denotes the rainfall event. Only data points where ΔRain is positive are displayed. “Acc.” (“Dec.”) refers to an increase (decrease) in *U*_*cloud*_, and “int.” (“sup.”) refers to an increase (decrease) in Rain_*p*95_. The quantity *N* is the percent of points falling in each quadrant and ΔMed indicates the median ΔRain value in each quadrant.

We conceptually separated two factors that are strongly associated with the initiation of flash floods: changes in high rainfall rates (ΔRain_*p*95_) and in rainfall advection speed (Δ*U*_*cloud*_)^51^. The former was defined as the difference in the 95^th^ percentile of accumulated rainfall over the urban grid points from the “urban” to the “no-urban” simulations. Figures 4 and S17 display the changes in both these properties and in the mean rainfall accumulation over the urban grid points (ΔRain) at each output time. Overall, 63% of the data points showed increased rainfall accumulations (ΔRain > 0), and the magnitude of the change was generally higher compared to the decreasing changes (Fig. S17), with median changes of 47% and −27%, respectively. This means that, across all rainfall events and output times, increased rainfall accumulation over the city occurred more frequently than suppression, and on average, the enhancement effect was stronger.

Figures 4 and S17 show a strong and positive relationship between ΔRain and ΔRain_*p*95_. Among the cases of rainfall increase (ΔRain > 0; Fig. 4), the largest proportion (59%, with a median increase of 55%) exhibited rainfall intensification effects (ΔRain_*p*95_ > 0) with a reduction in advection speed (Δ*U*_*cloud*_ < 0). An additional 26% (with a median increase of 51%) of the rainfall increase cases featured patterns of intensification and speed increases, 9% with patterns of suppression and slowing, and the remaining 6% showed patterns of suppression and speed increases. These results indicate that the urban-induced intensification of rainfall is the main driver of the increases in rainfall accumulation over the city; however, when intensification occurred together with advection slowing, the rainfall enhancement was more frequent and its magnitude was larger.

## Discussion

We have investigated the changes in the speed of convective rainfall events using radar observations across eight urban areas and numerical simulations of 10 events over Indianapolis. The analysis of weather radar data revealed that the motion of convective rain cells decelerated over five cities (Birmingham, London, Indianapolis, Charlotte, and Atlanta); these were among the largest (ranging from 625 to 6340 km^2^), and the effect was the strongest over and downwind of the cities (Fig. 1a). It is worth noting that three cities (Milan, Berlin, and Phoenix) did not show significant changes in rain cell speeds. Differences in the geographical setting, background climate, as well as the strength and extent of the dynamic and thermodynamic effects of the urban area on the local climate may explain the observed differences. For example, in the case of Phoenix, although the urban area is generally flat, it has some topography in its surroundings. In this city, wind flow patterns may be controlled by topographic circulations or their interaction with the urban influence^58^. Further research is needed to better understand inter-city differences in storm speed changes and the associated mechanisms.

Ten heavy rainfall events were then simulated and analyzed for Indianapolis, where an urban-induced slowed advection is observed, using a convection-permitting model. Six of the simulations revealed a reduction in the advection speed of rainfall by showing decreased horizontal wind speeds at the cloud layer over the cities (Fig. 2a). Although some studies have indicated that the drag effect from increased surface roughness may affect both surface and planetary boundary layer wind patterns, as well as rainfall motion^36^, here we did not find evidence that roughness effects extended to the cloud layer and therefore decelerated the cloud-layer winds. In many of the simulations, we observed that deceleration effects due to the rough urban surface extended from the surface upward until 1 or 2 km, but not reaching the cloud layer (visible from the cross-sections; Figs. 3, S10, S12, S14, S15 and S16). Since in our simulations Indianapolis was mostly represented by openly spaced, low-rise buildings (LCZ 6) with an average height of 6.5 m, stronger building-barrier effects may be expected in cities with taller or denser urban structures.

**Fig. 5:**
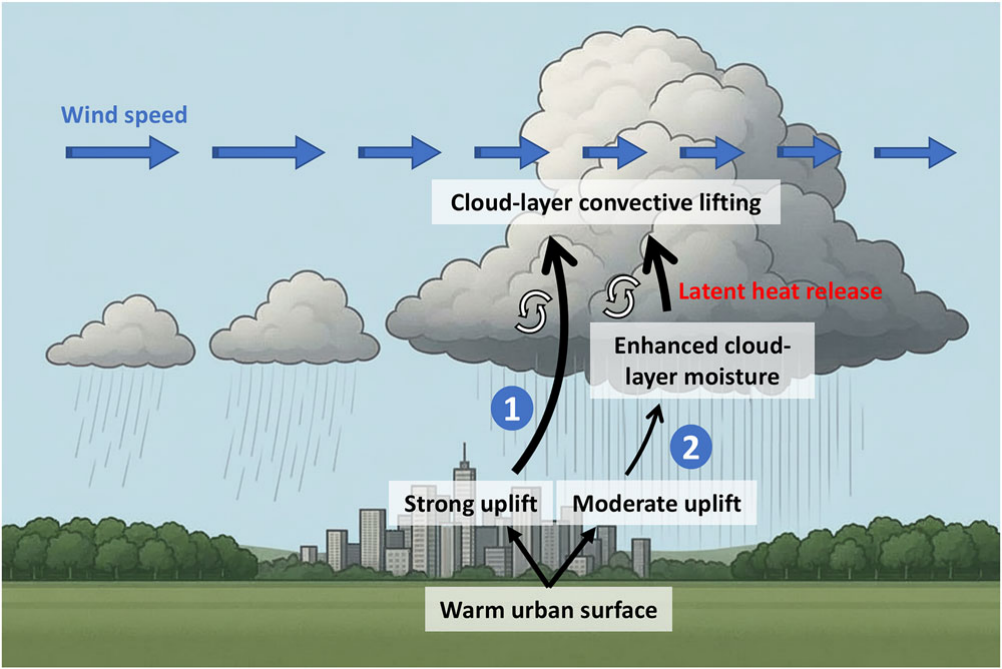
Schematic summarizing the key processes involved in the urban-slowing of heavy rainfall. In all rainfall events that showed a slowing effect in the “urban” compared to the “no-urban” simulations, the urban surface was consistently warmer. In several of these cases, this enhanced surface heating led to strong vertical motion above the urban area that extended to the cloud layer. This occurred for events C, D, E and I, and is illustrated as process 1. In other simulations, low-level uplift was present but weaker, and convective lifting at the cloud layer was triggered by increased humidity at the cloud layer, which through latent heat release lead to increased buoyancy. This occurred for events B, F, and G, and is illustrated as process 2. In both cases, the cloud-layer convective lifting transported horizontal momentum vertically, reducing the horizontal advection speed of rainfall.

Rather than being driven by the dynamical roughness effects of the city, the cloud-layer wind speed changes were instead linked to thermodynamic effects induced by the urban area; specifically, to changes in (upward) vertical wind speeds over the urban area compared to the “no-urban” simulations (Fig. 2b). Rising motion over an urban area may occur due to convective lifting from the thermal gradient between an urban area and its surroundings^59^, low-level horizontal convergence resulting from the UHI circulation^60^, or due to mechanical uplift effects arising from increased aerodynamic roughness^61^. We found that these first two mechanisms explained the observed changes in horizontal cloud motion. Four of the simulated events (C, D, E, and I) presented a well-developed UHI near the surface, along with low-level horizontal convergence over the city and increased uplift above it. This uplift then extended upward to the atmospheric layers containing the cloud, where a general decrease in speed at the cloud layer was observed. For the three other slowing events (B, F, and G), which also occurred with a UHI present, low-level wind convergence and uplift in the lower atmosphere were not as pronounced. Instead, areas of maximum vertical motion were confined to higher levels of the atmosphere, around 3–5 km, where the clouds were located. In these layers, strong uplift was driven by surface instability, which was weaker but still present. Increased uplift over the city then resulted in enhanced cloud-layer humidity (*Q*_*cloud*_, Figs. S8f, S11f and S12f), leading to latent heat release, warmer cloud temperatures, and enhanced convective lifting within the cloud, as also observed in Li et al.^24^. Figure 5 provides a summary of these mechanisms. Overall, our results are in agreement with other studies that have suggested that increased atmospheric instability and vertical mixing from a warm surface can potentially decrease horizontal wind speeds within the urban boundary layer^47,49^. We anticipate that the increased vertical motion from the stronger updrafts results in the vertical transport of horizontal momentum, thereby reducing horizontal winds. This type of convective momentum transport has been observed in mesoscale convective systems, where horizontal wind speeds decrease in regions of strong convection^62,63^.

Next, we discuss the potential contribution of the slowing of convective storms to the urban flood hazard. Intensification of the heaviest fraction of the rainfall was found to be the primary driver of increased rainfall over the city (Figs. 4 and S17). We noticed more and stronger cases of increased rainfall accumulation when rainfall intensification occurred at the same time as advection speed decreases. While the tendency of cities to intensify rainfall and consequently increase flood hazards has been examined and discussed^10,18,64^, our results suggest that urban-induced slowing of rainfall advection over cities likely contributes to further increasing this hazard, and that urban rainfall enhancement and slowing down are primarily coupled processes (Fig. 4). Our findings imply that both the intensification and slowing down of rainfall may arise from the same underlying mechanism, specifically the increased uplift induced by the urban area.

One factor not accounted for in our radar data analysis is the potential influence of urban areas on storm life cycles and their effect on rain cell movement speed. Urban areas are known to increase the frequency of storm initiations over them^27,65^ or affecting processes such as storm merging and splitting^25,26^. Consequently, this may also be influencing the climatological speed patterns. However, the numerical simulation analysis has demonstrated that urban areas can dynamically slow down rainfall advection, suggesting that the effects are not solely due to these life cycle changes. While numerical experiments that compare rainfall simulations over urban and non-urban areas are widely used to study urban rainfall signatures [e.g., refs. ^6,10,29^], this approach has some limitations. For example, model outputs may be sensitive to initial atmospheric conditions and the physical parameterization schemes used^66–69^, which can introduce uncertainty in the quantification of the urban effect^70^. Therefore, addressing such uncertainty with ensemble modeling approaches provides opportunities to determine more robust urban signals [e.g., refs. ^34,71,72^]. Furthermore, our findings suggest that a better understanding of the conditions driving rainfall slowing is needed. For example, we found that longer rainfall residence times may be associated with stronger urban influences on rainfall. This association could arise because slower-moving rainfall systems or larger cities prolong the time rainfall spends over an urban area, increasing the potential for urban-induced perturbations^73^.

To summarize, we have explored how urban areas impact the advection speed of heavy rainfall, which is a key rainfall property for flood hazard^50–56^, and remains largely unexplored. Our results have shown that storm speed is often reduced over cities, mostly because of thermodynamic factors. The effect is more pronounced in large cities and frequently occurs alongside heavy rainfall intensification. This indicates that there is a potential for heavy rainfall duration to be lengthened over cities, ultimately increasing storm rainfall volumes. Although our results also displayed cases of rainfall movement acceleration over some cities or simulations, this does not contradict the broader implication that urbanization can contribute to increasing flood hazards. The intensification and slowing of even a subset of rainfall events, rather than all events, can still lead to a significant increase in flood risk, especially given that the events studied were rainfall extremes. As urban areas are expected to become more populated in the future^2^, we must deepen our understanding of the components and mechanisms that affect the intensification and slowing down of urban heavy rainfall to better forecast its impact on future cities.

## Methods

### Study cities

The studied urban areas are located in Europe (Milan, Italy; Berlin, Germany; and London and Birmingham, United Kingdom) and the United States (Phoenix, Arizona; Charlotte, North Carolina; Atlanta, Georgia; and Indianapolis, Indiana). These cities were selected because they had good-quality radar data available and were located in areas that are generally less affected by other rainfall-influencing factors, such as large bodies of water, mountainous terrain, or proximity to other large urbanized areas, or where the impact of these factors is expected to be small. Additionally, the cities were located in different geographical regions and represented a variety of urban forms, based on their Local Climate Zones (LCZ)^74^. These eight urban areas have also been shown to modify heavy rainfall properties, mainly by intensifying it and changing its spatial structure^27^. Maps of their LCZs, produced with data from Demuzere et al.^75–78^, are shown in Fig. S1. The boundaries of the urban areas were defined following urban area classifications from regional agencies^79,80^.

### Weather radar data and analysis

The radar data analysis was performed with data from 7 years (2015–2021) for the summertime months only (May to September), when the studied cities experience heavy rainfall. We used composite weather radar data products of rainfall rate, which were quality-controlled and provided by the data agencies listed below. For the U.S. cities, we used data at 1 km and 4 min resolution, obtained from the Multi-Radar Multi-Sensor Surface Precipitation Rate product from the National Centers for Environmental Prediction^81^. These data are from a network of S-band radars and are corrected with numerical weather prediction model data. For the European cities, the data used was at 1 km and 5 min resolution and was from networks of C-band weather radars. We used the fourth-generation weather radar system of MeteoSwiss^82^ for Milan, the Radar Climatology (RADKLIM) data set^83^ from the German Weather Service (DWD) for Berlin, and the Met Office’s NIMROD product^84^ for the UK cities. The weather radar data obtained in this study were identical to those used in Torelló-Sentelles et al.^27^.

We followed the methodology of Torelló-Sentelles et al.^27^ to track heavy rainfall events and extract different rainfall properties. The Iterative Rain Cell Tracking algorithm (IRT, Moseley et al.^85^) was used to track heavy rainfall events. A threshold of 10 mm h^−1^ for intensity and 9 km^2^ for area was used to identify rainfall objects. The IRT then linked these objects to different convective precipitation tracks. A threshold of 15 min was applied to filter out unrealistic tracks with a short lifetime. The position of each object relative to the city centers was determined by extracting the distance from the city center to each object’s weighted center of mass, and establishing if it was upwind or downwind of the city center by considering each track’s mean direction of motion. All rainfall objects were included in our analysis, regardless of whethertheir tracks passed over the city. Rain cell movement speed was computed by the IRT algorithm at each time step by calculating the distance between the weighted center-of-mass positions of the rain cell at two consecutive time steps.

Three urban bins (urban, upwind, and downwind) and an upwind control bin were created for each urban area. The footprints of the bins were relative to the size of each city and were rotated according to each track’s mean direction of motion. The urban bin extended up to 0.5 times the average city radius (R). The upwind and downwind extended from 0.5 to 1.5 R, and the control from 1.5 to 2 R. Figure S2 illustrates the binning setup for one rainfall track. Changes in median rain cell speed and mean rainfall intensity (Figs. 1 and S3) were computed at each bin, relative to the control bin. The control bin was assumed to be sufficiently distant from the city to remain unaffected by it, but placed not far enough away from the city for rainfall characteristics to be altered by regional effects, such as differences in land cover, land use, or topography. A non-parametric permutation test was used to test for statistical significance of the median changes^86^, and the confidence intervals were computed using bootstrap resampling. We tested different distance thresholds for the bins and found that, although the bin positioning sometimes affected the magnitude of rainfall property changes, the overall patterns across cities remained consistent.

A composite map of mean object speed was created by: (i) obtaining each rainfall object’s estimated movement speed from the storm-tracking algorithm, (ii) assigning this speed value to each grid point pertaining to that object, (iii) rotating each rainfall object around the city center based on its track’s mean direction of motion, and (iv) computing the composite mean of all the rotated objects. Objects with speed values over 30 m s^−1^ were removed from this analysis to remove rain cells with unrealistic speeds. Figure 1b shows the deviation from the composite mean across all grid points for Indianapolis and the maps for the other cities are presented in Figs. S4 and S5. The object rotation was performed to assess the changes relative to the city’s upwind and downwind regions. In this rotated frame, the eastern and western regions correspond to the downwind and upwind areas, respectively. Regions north and south of the city center may include objects that did not cross the city but moved laterally past it, as well as contributions from large rain cells that partly overlapped the urban area.

We note that our radar data analysis is subject to some limitations. For example, storm motion may also be influenced by factors other than the urban area, such as potential radar artifacts, heterogeneities of the land use/land cover in some urban surroundings, or, in the cases of Milan and Phoenix, their relative proximity to areas of higher elevation, especially in the control bins. In addition, uncertainties may stem from the rainfall tracking algorithm when identifying and tracking rainfall objects throughout their lifetime, and during classification of merging and splitting events. Inaccuracies in rain cell velocities may also arise because rain cell intensity fields are often heterogeneous and irregularly shaped, which can complicate the determination of their boundaries and center point, and consequently, their movement. However, such uncertainties are not expected to have a major influence on our analysis, as potential misclassifications should occur randomly across the study domain, thus not introducing systematic biases between urban and rural areas.

### WRF simulations

The convection-permitting simulations were for the city of Indianapolis (Figs. S1 and S6). This city was selected for our analysis because it displayed both storm intensification and deceleration within the city in the weather radar data analysis (section “Results”), and relative to all the study cities, it has a strongly homogeneous surrounding land cover (dominated by croplands), the terrain is flat, and is not near other large urban clusters.

We numerically simulated 10 convective summer rainfall events that crossed the city using the Weather Research and Forecasting (WRF) model (version 4.3.3)^87^. These events were chosen because of their weak synoptic forcing, which was estimated by using the convective adjustment time scale^88–90^, and as WRF simulates them well^91^. The numerical simulations were set up as the ones performed for the city of Indianapolis in ref. ^91^, who evaluated changes in rainfall intensity as a result of the urban area’s presence. Figure S6a shows the model domain setup. The outer and inner domains had a horizontal grid spacing of 9-, 3-, and 1-km, respectively. The RRTMG shortwave and longwave schemes^92^, the single-moment 6-class microphysics scheme (WSM6)^93^, the revised MM5 scheme^94^, the Noah-MP land surface model^95,96^, the Kain-Fritsch scheme (for the 9-km domain only)^97^, and the Building Environment Parameterization^98^ were used. The urban land use and cover were represented using WUDAPT-derived LCZ maps, which provide information on typical urban morphological characteristics^99^. Boundary and initial conditions, with a horizontal grid spacing of 0.25°, were acquired from the hourly ERA5 climate reanalysis product^100^. The model output frequency was set to 15 min and the data used for the analysis was from the inner 1-km domain. The simulations were evaluated against rainfall data obtained from weather radars that were bias-corrected using ground gauges^81^ and are reported in ref. ^91^.

Relevant information about the simulated rainfall events is summarized in Table S1. Each rainfall event was simulated under two scenarios: an “urban” case using the current land use/land cover of Indianapolis (Fig. S6c), and a “no-urban” case where the city’s land use was replaced with the dominant surrounding land cover (Fig. S6b), with this being the only modification in the model. The “no-urban” land use/land cover maps were created using the WUDAPT-to-WRF (W2W) package^99^. Time series of accumulated rainfall within the urban area for both scenarios are displayed in Fig. S18.

To quantify the storm speed from the simulations, we computed the mean horizontal wind speed averaged over: all vertical levels, urban grid points, output times where the mean accumulated rainfall over the urban area was above 0.025 mm per 15 min, and grid points (in both the horizontal and vertical directions) where the cloud water mixing ratio (*Q*_*cloud*_) was above 0.05 g kg^−1^. The mean accumulated rainfall threshold was imposed so that output times without substantial rain over the city were excluded. This computed variable, hereafter named *U*_*cloud*_, served as a proxy for rain-producing cloud movement speed. Here, we assumed that advection controlled the movement of convective cells since the mean winds in the tropospheric layer in which clouds are situated tend to be strongly related to convective cell movement^51,101^. Cloud-layer wind speeds were used as an alternative to rain cell tracking in the simulation analysis because, at the coarser 15-min temporal resolution (compared with 4 or 5 min for the radar), rainfall could not be tracked satisfactorily, and estimates of individual storm movement were often noisy.

We computed the differences in accumulated rainfall and the medians of *U*_*cloud*_ between the “urban” and “no-urban” simulations over the urban area for all rainfall events. Both variables are plotted in Fig. 2a. Next, to explore whether enhanced vertical motion over the urban area was related to changes in horizontal cloud-layer wind speeds, we computed the positive vertical wind speeds (*w*_*pos*_) over the urban area, also considering values across all vertical levels and output times where the mean accumulated rainfall over the urban area was above 0.025 mm per 15 min. In Fig. 2b, we plotted the difference in the medians of *U*_*cloud*_ and *w*_*pos*_, also between the “urban” and “no-urban” simulations. A simple linear regression analysis was performed to quantify the relationship between changes in *U*_*cloud*_ and *w*_*pos*_. We also explored links between *U*_*cloud*_ differences and other model variables, including boundary layer temperature differences, background wind speed, peak rainfall time, and rainfall residence time over the city. Data for the latter two variables are displayed in Table S1. Peak rainfall time was defined as the time at which the rainfall, averaged over the urban area in the “urban” simulation, reached its maximum. Residence time was computed as the total period of time during which the mean accumulated rainfall over the urban area exceeded 0.025 mm, in the “urban” simulation.

The mechanisms related to *U*_*cloud*_ changes were further explored by analyzing area-averaged cross-sections of different convection-related variables. Eleven vertical cross-sections, oriented to match the mean direction of the horizontal winds at 500 hPa from the “urban” simulation over the inner 1-km domain, were taken within an area extending 5 km from the city center in each direction perpendicular to the cross-section’s direction. The dashed lines in Fig. S7 display the positions of the cross-section lines for each rainfall event. An average of these multiple cross-sections was then taken per rainfall event. We explored the cross-sections of: *Q*_*cloud*_ and the change, defined as the difference between the “urban” and “no-urban” case in, horizontal wind speeds (ΔU), positive vertical wind speed (Δ*w*_*pos*_), temperature (Δ*T*), convergence of winds (ΔCon), and the wind field difference. All the cross-sections were averaged over the simulation output times during which the mean accumulated rainfall over the urban area exceeded 0.025 mm per 15 min in both the “urban” and “no-urban” simulations.

Last, we examined the potential effects of rainfall intensification and reduced advection speed on accumulated rainfall over the urban area. Rainfall intensification was defined as the change in the 95^th^ percentile of accumulated rainfall at each 15-min output time between the “urban” and “no-urban” simulations (ΔRain_*p*95_). This variable quantified the changes to the high rainfall rates. In Fig. 4 we display ΔRain_*p*95_, the changes in mean *U*_*cloud*_ (Δ*U*_*cloud*_), and the changes in mean accumulated rainfall over the urban area (ΔRain), for all output times and rainfall events. All of these variables were computed per simulation output time (every 15 min) and output times where the mean accumulated rainfall over the urban area was below 0.025 mm per 15 min were excluded from this analysis as well.

There are some limitations in the WRF modeling framework, including uncertainties in how the model parameterizes the urban environment and urban-related atmospheric processes. Yet, WRF is a common tool to simulate urban rainfall [e.g., refs. ^7,10,24,31,34,61,91^]. For example, although we ran the simulations at relatively high resolution (1 km horizontal grid spacing), the grid spacing is still too coarse to fully resolve individual updrafts and downdrafts, introducing some uncertainty in the representation of convective processes. In addition, although we used maps of LCZs at 1 km resolution to represent the urban land use and land cover, this representation is still more homogeneous than the actual urban fabric. Cities exhibit considerable sub-kilometer heterogeneity in building morphology, land cover, and surface properties, therefore, some local-scale processes may not be fully represented in the model. We note that our analysis focused on rainfall and convection-related variable changes throughout the entire rainfall event and across the urban area, which may have overlooked localized processes occurring at smaller spatial and temporal scales. Furthermore, our quantification of rainfall slowing, based on differences between the “urban” and “no-urban” simulations above the city, may partly reflect changes in rainfall patterns unrelated to deceleration, such as shifts in the spatial location of rainfall. In addition, since our study concentrated on the urban area, the effects occurring in the city peripheries, such as the lateral sides of the city or downwind regions where airflow convergence has been reported in some studies^37,102^, were not quantified.

## Supplementary information

Supplementary Information

## Data Availability

The following weather radar data sets were used in the radar data analysis: the fourth-generation weather radar system of MeteoSwiss^82^; the RADKLIM quasi gauge-adjusted 5-min precipitation rate from the Deutscher Wetterdienst (DWD)^83^; the 1 km Resolution UK Composite Rainfall Data from the Met Office Nimrod System^84^ via the NCAS British Atmospheric Data Centre^103^; and the Multi-RADAR Multi-Sensor (MRMS) Archiving^81^ via the Iowa Environmental Mesonet^104^. We obtained the following urban boundary data sets: the administrative boundary data sets for European study areas for 2020 from Eurostat^80^, and the 2020 U.S. urban area delineations from the Census Bureau^79^. The rainfall data product “GaugeCorrQPE01H,” which was used to validate the WRF simulations, is available from the Multi-Radar Multi-Sensor (MRMS) archive^81^ at the Iowa Environmental Mesonet from Iowa State University (https://mtarchive.geol.iastate.edu/).
