# Supplementary material for: When storms slow down: urban effects on rainfall accumulation and flood hazard
Source: NPJ Nat Hazards. 2025 Dec 20;2(1):106. doi: 10.1038/s44304-025-00158-4 (PMC12718176; doi:10.1038/s44304-025-00158-4)
Supplement: Supplementary file 1 — Supplementary Information [file 44304_2025_158_MOESM1_ESM.docx]

**Supplementary Information for “When storms slow down: Urban effects on rainfall accumulation and flood hazard”**

Herminia Torelló-Sentelles^1^, Marika Koukoula^1^, Gabriele Villarini^2,3^, Francesco Marra^4^, Nadav Peleg^1,5^

^1^Institute of Earth Surface Dynamics, University of Lausanne, Lausanne, Switzerland

^2^Department of Civil and Environmental Engineering, Princeton University, Princeton, USA

^3^High Meadows Environmental Institute, Princeton University, Princeton, USA

^4^Department of Geosciences, University of Padova, Padova, Italy

^5^Expertise Center for Climate Extremes, University of Lausanne, Lausanne, Switzerland

**
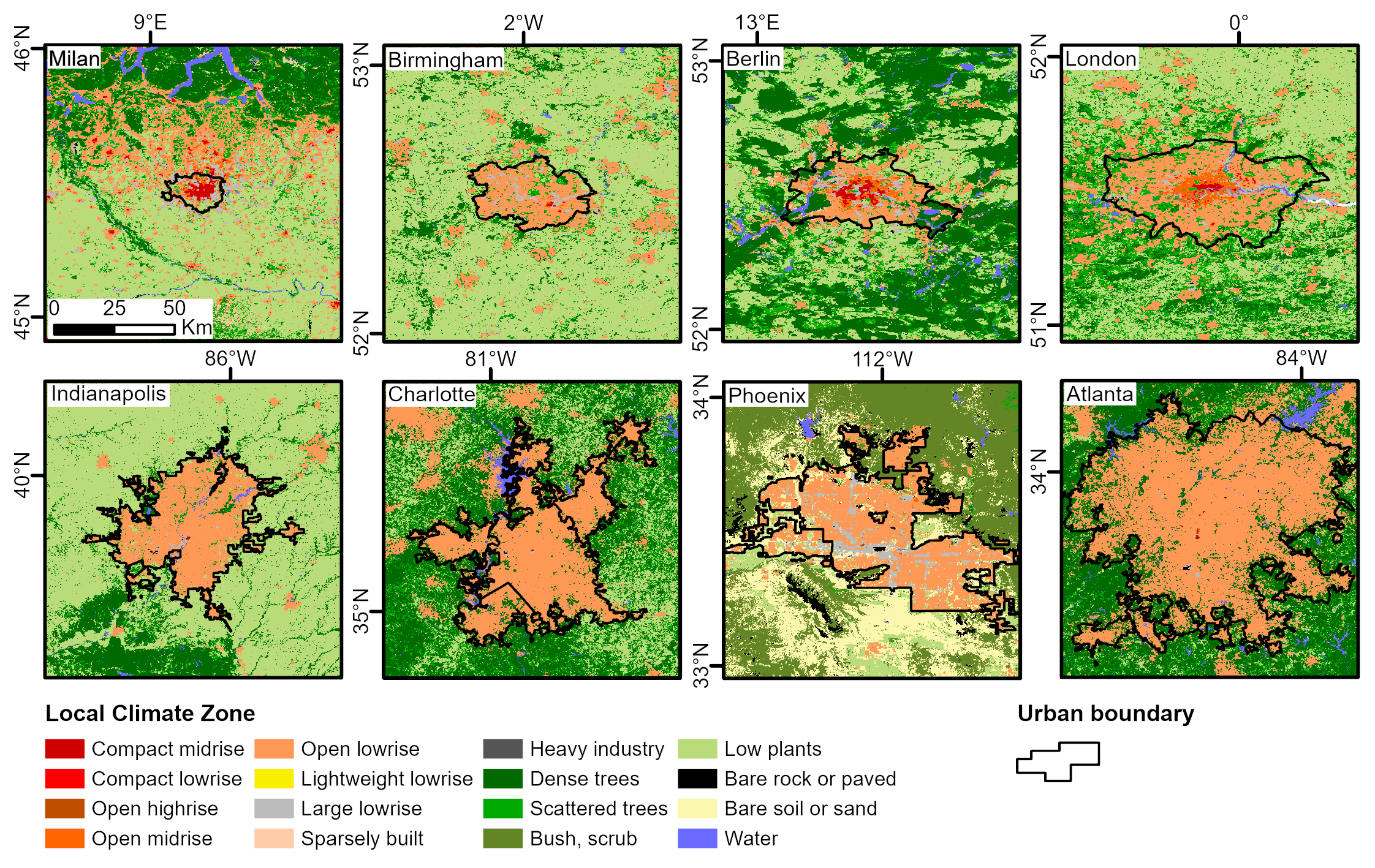
Figure S1.** Local Climate Zone (LCZ) maps for different cities, ordered by their size. The maps use a consistent scale for comparison. Urban boundaries are delineated by black lines. This figure is adapted from [1].


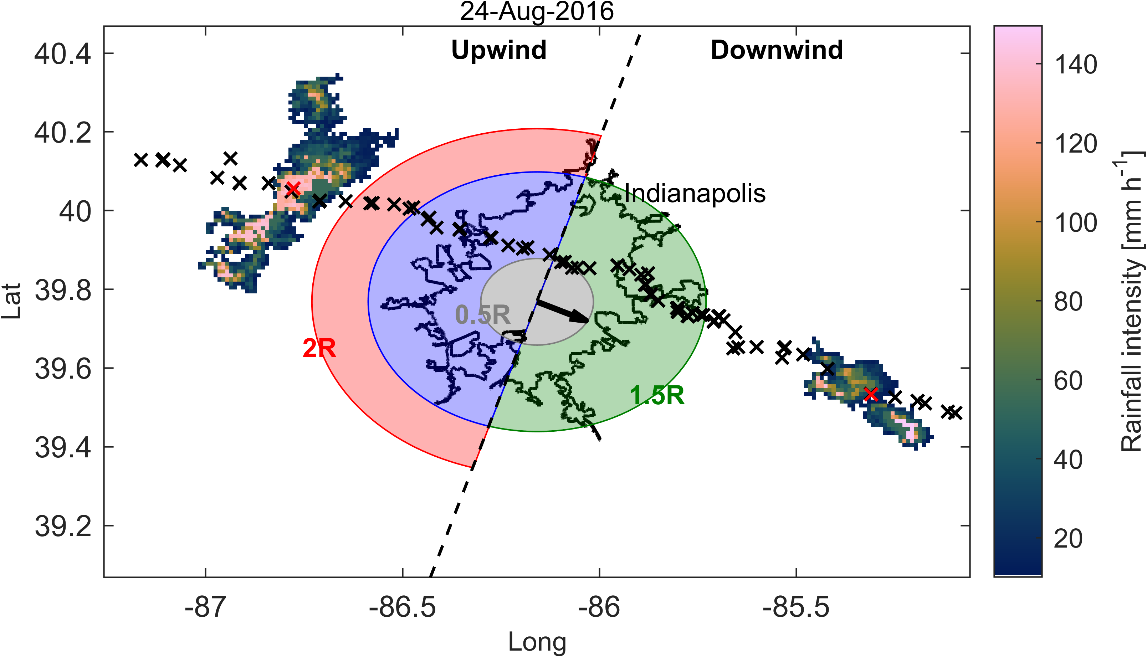


**Figure S2.** Illustration of the binning approach used in the radar data analysis. The trajectory of one track is displayed. The weighted center of mass of each rainfall object is marked with a cross and the mean direction of motion of the track is displayed in a black arrow. Two rainfall objects, one near the start and end of the track’s lifetime are shown. The weighted center of mass of each is marked in a red cross. The control bin is shown in red and extends from 2 to 1.5 times the average city radius (*R*), the upwind (blue) and downwind (green) bins extend from 0.5 to 1.5 *R*, and the urban (grey) bin extends from the city center to 0.5 *R*. All four bins are rotated according to each track’s mean direction of motion. The urban boundary of Indianapolis is shown in a black polygon.


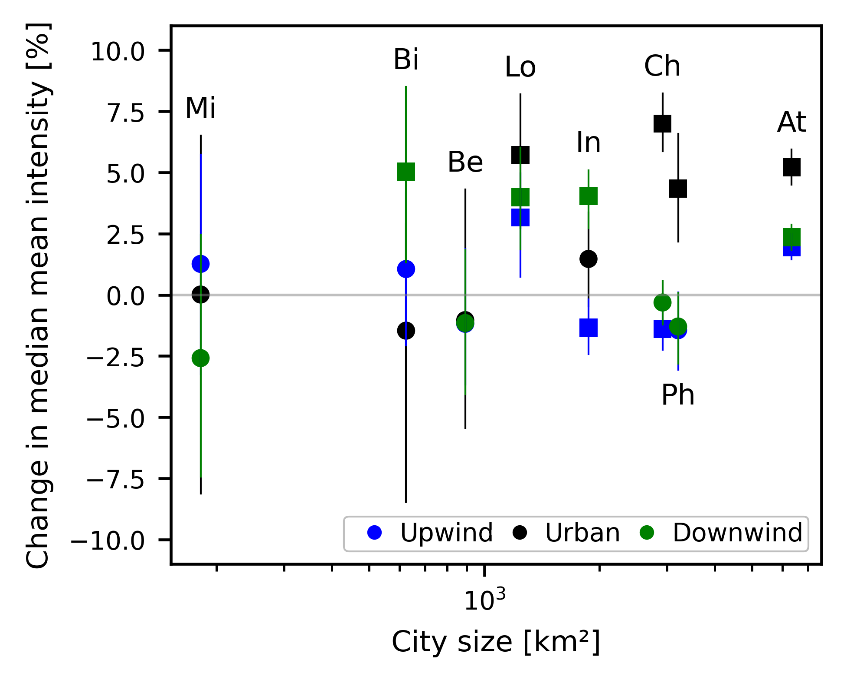


**Figure S3.** Change in median rainfall object mean intensity from the control to the three urban bins, plotted against city size. The color of the points refers to the urban bin and the error bars represent 95% confidence intervals of the median differences. If the points are square (round), then the median change was (not) significant (*p* < 0.05).


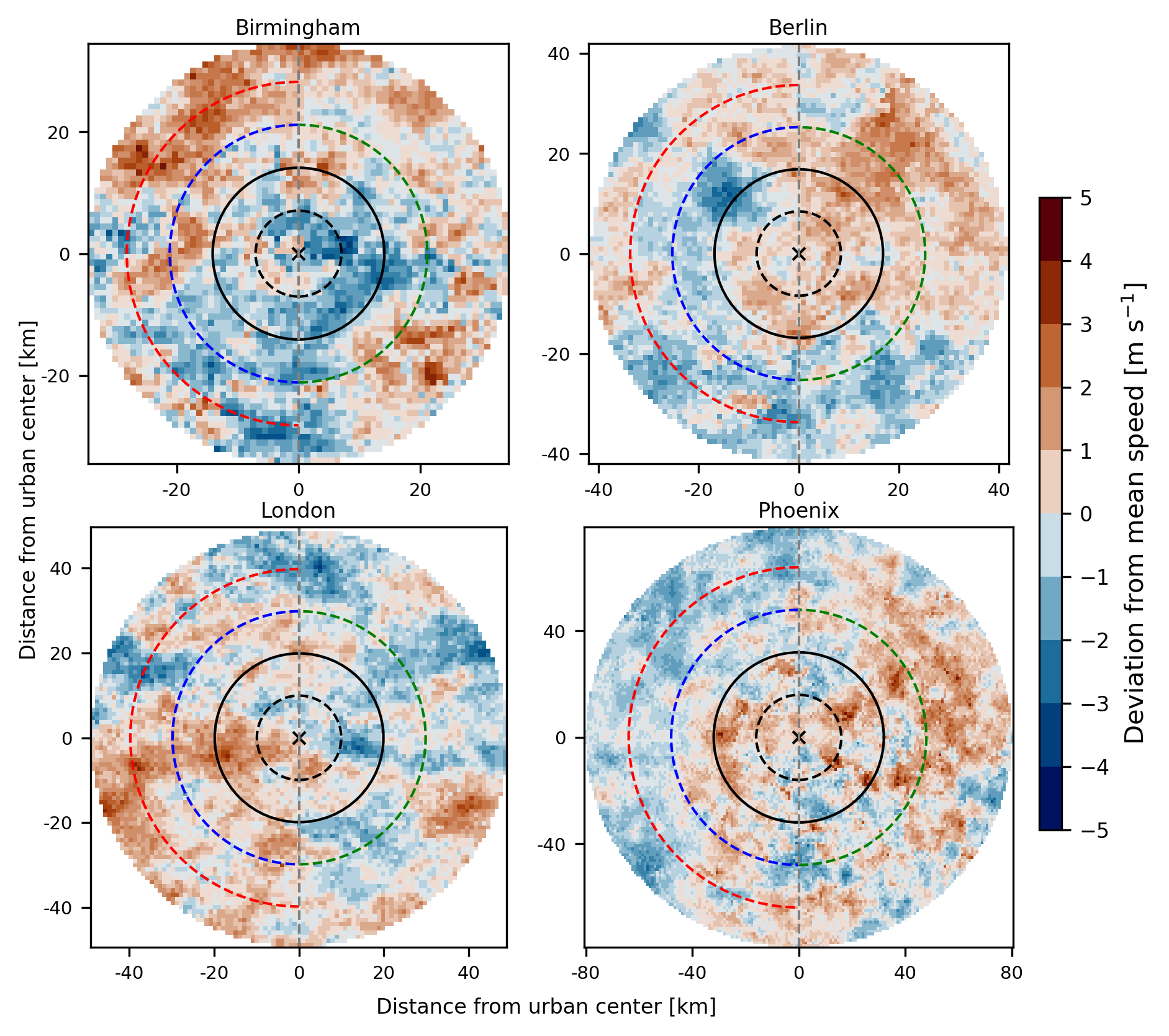


**Figure S4.** Composite of mean rainfall object speed over different study areas, expressed as the deviation from the mean. Each rainfall object is rotated around a fixed center located at each city’s center, based on the corresponding track's mean direction of motion. For all tracks, the downwind direction is to the east, and the upwind direction to the west. The black circle shows the average radius of the city, and the city center is marked with a cross. The black, blue, green, and red dashed lines indicate the edges of the urban, upwind, downwind, and control bins, respectively.


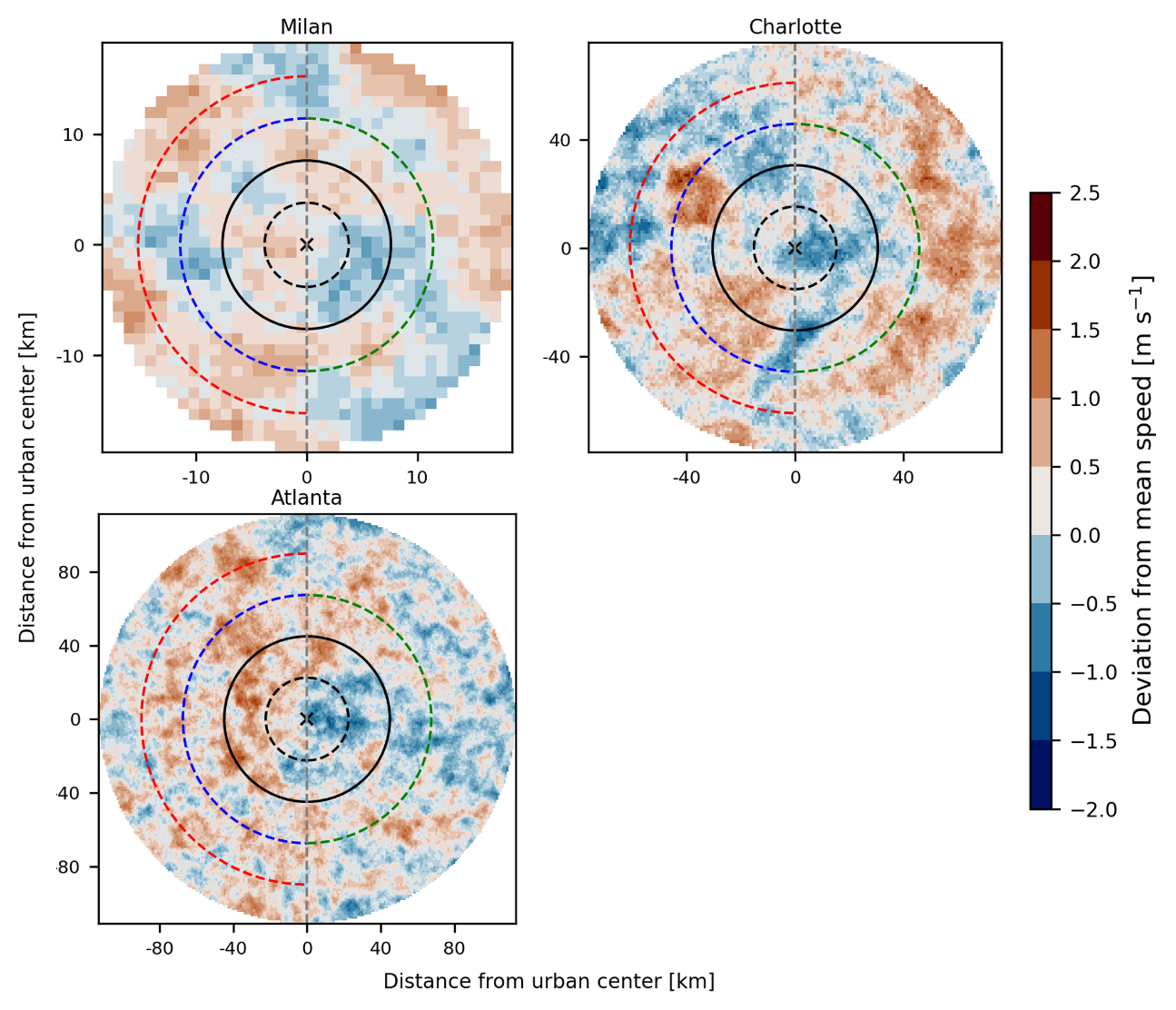


**Figure S5.** Composite of mean rainfall object speed over different study areas, expressed as the deviation from the mean. Each rainfall object is rotated around a fixed center located at each city’s center, based on the corresponding track's mean direction of motion. For all tracks, the downwind direction is to the east, and the upwind direction to the west. The black circle shows the average radius of the city, and the city center is marked with a cross. The black, blue, green, and red dashed lines indicate the edges of the urban, upwind, downwind, and control bins, respectively.

**
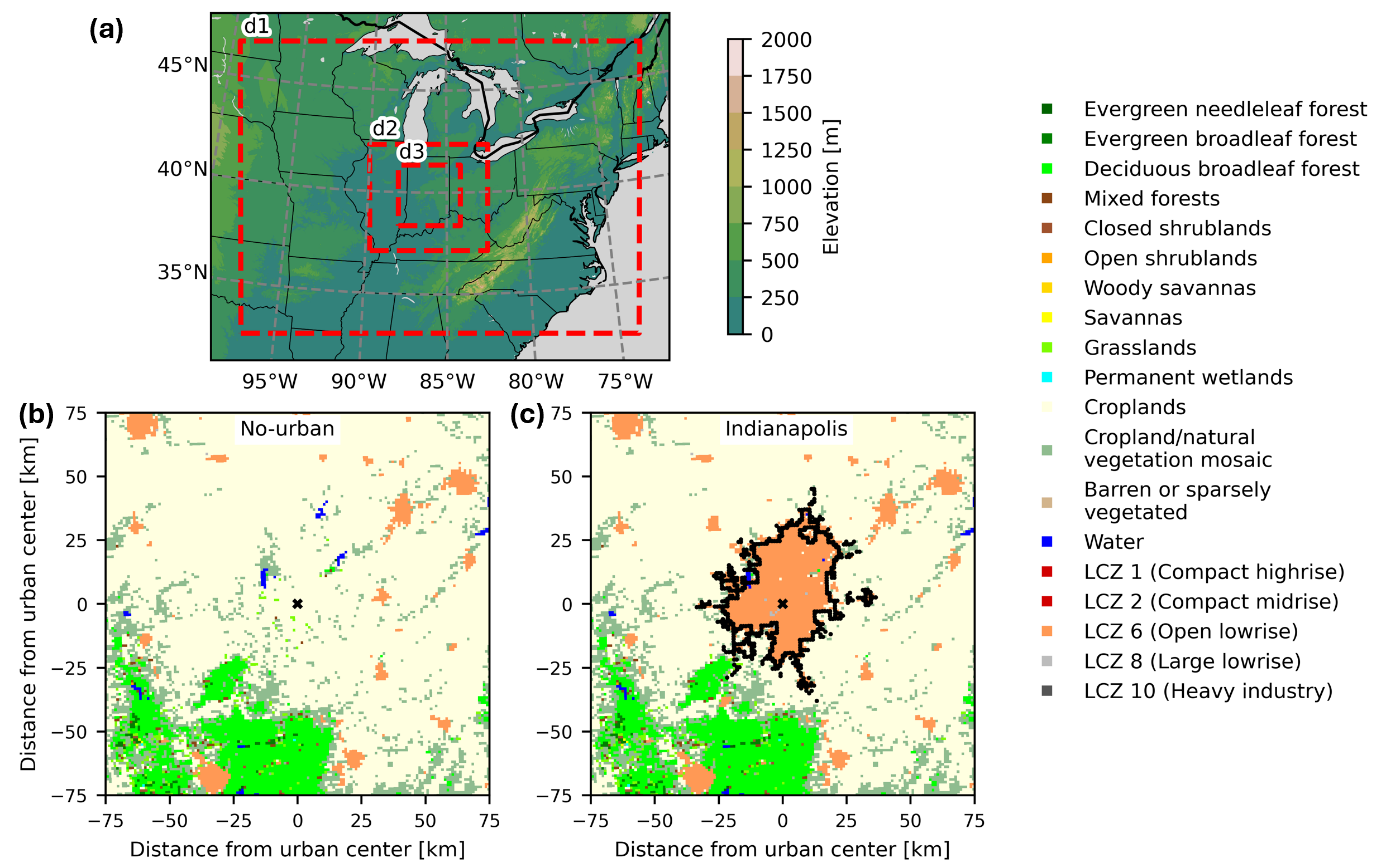
Figure S6.** (a) WRF domain setup and elevation map, centered over the city of Indianapolis. The three model domains (d1, d2, and d3) are outlined with dashed red lines. (b) and (c) show land use/land cover maps used in the WRF simulations for the 'no-urban' and 'urban' scenarios, respectively. The cross marks the city center, and the black outline indicates the urban boundary. The plots are adapted from [2].


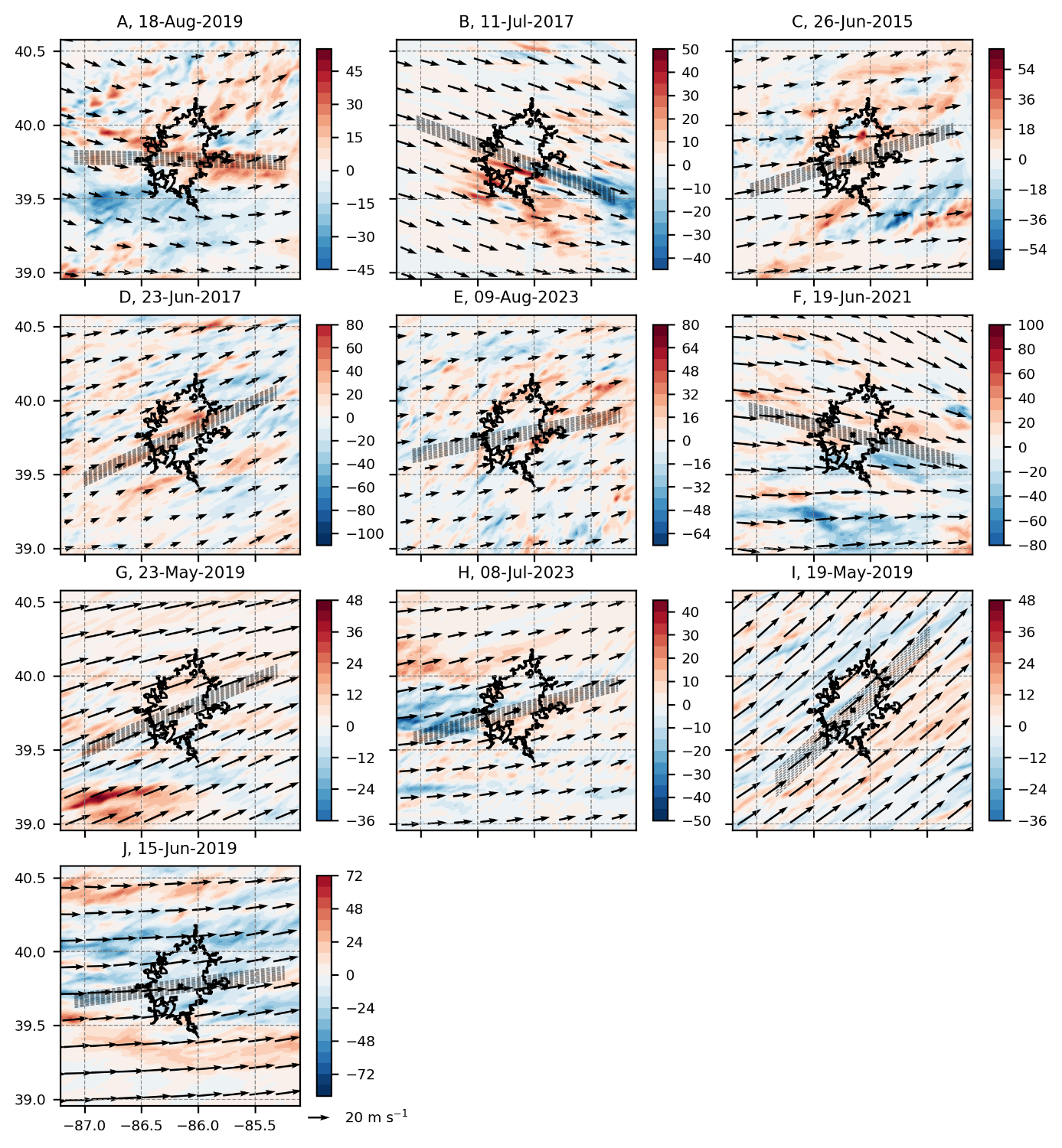
**Figure S7.** Maps of change in accumulated rainfall (mm) from the ‘urban’ to ‘no-urban’ simulations. Vector arrows show the mean horizontal winds at 500 hPa from the urban simulation, averaged over all output times between the start and end of each event. The dashed lines indicate the positions of the 11 cross-sections used in the analysis, which are oriented to match the mean direction of the horizontal winds at 500 hPa from the `urban' simulation. The urban boundary of Indianapolis is outlined in black.


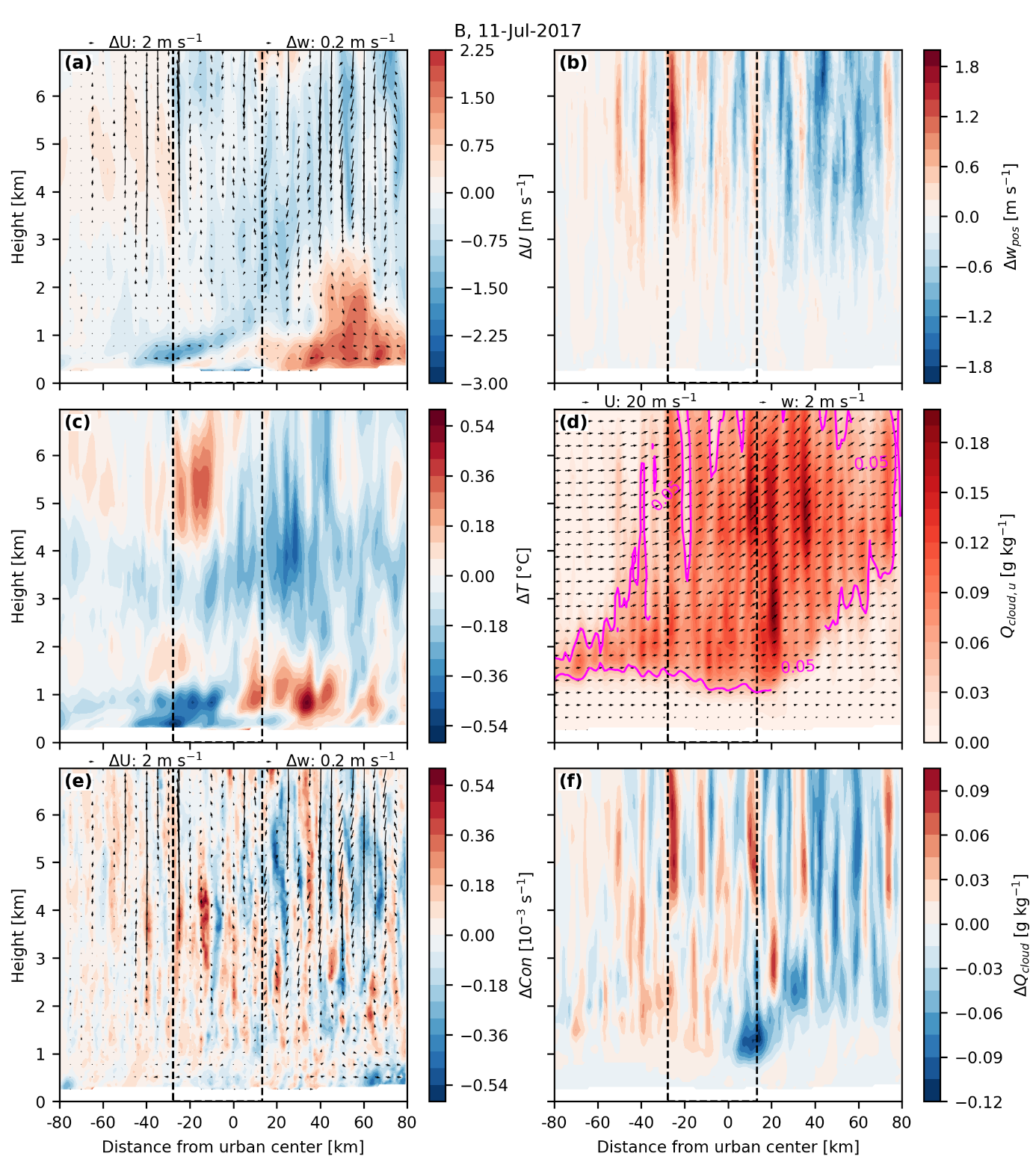
**Figure S8.** Area-averaged vertical cross-sections of several convection-related variables between the ‘urban’ and ‘no-urban’ simulations of Event B. Changes are presented for (a) *U*, (b) *w_pos_*, (c) *T*, (e) *Con*, and (f) *Q_cloud_*, (d) Cloud water mixing ratio (*Q_cloud_*) from the ‘urban’ simulation, with magenta contours indicating values of 0.05 g kg^−1^. In (d), vector arrows represent the wind field, with the vertical component indicating vertical motion and the horizontal component representing wind speeds along the cross-section line. In (a) and (e), vector arrows show the difference in the wind field between the ‘urban’ and ‘no-urban’ simulations. Note that vertical vectors are scaled by a factor of ten relative to the horizontal direction. The dashed black lines indicate the extent of the urban area. Distances from the urban center are negative when upwind and positive when downwind.


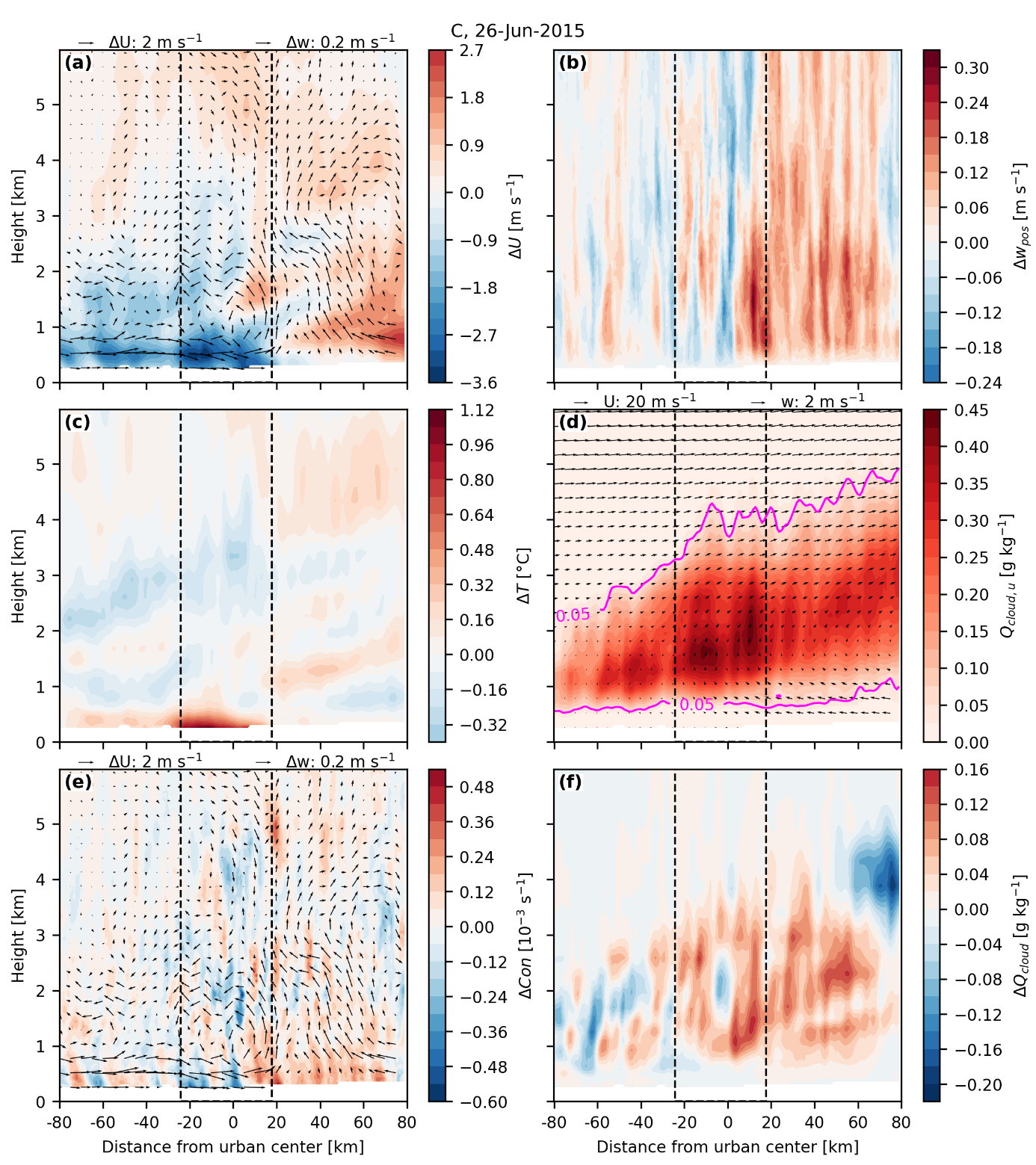
**Figure S9.** As Fig. S8, but for event C.


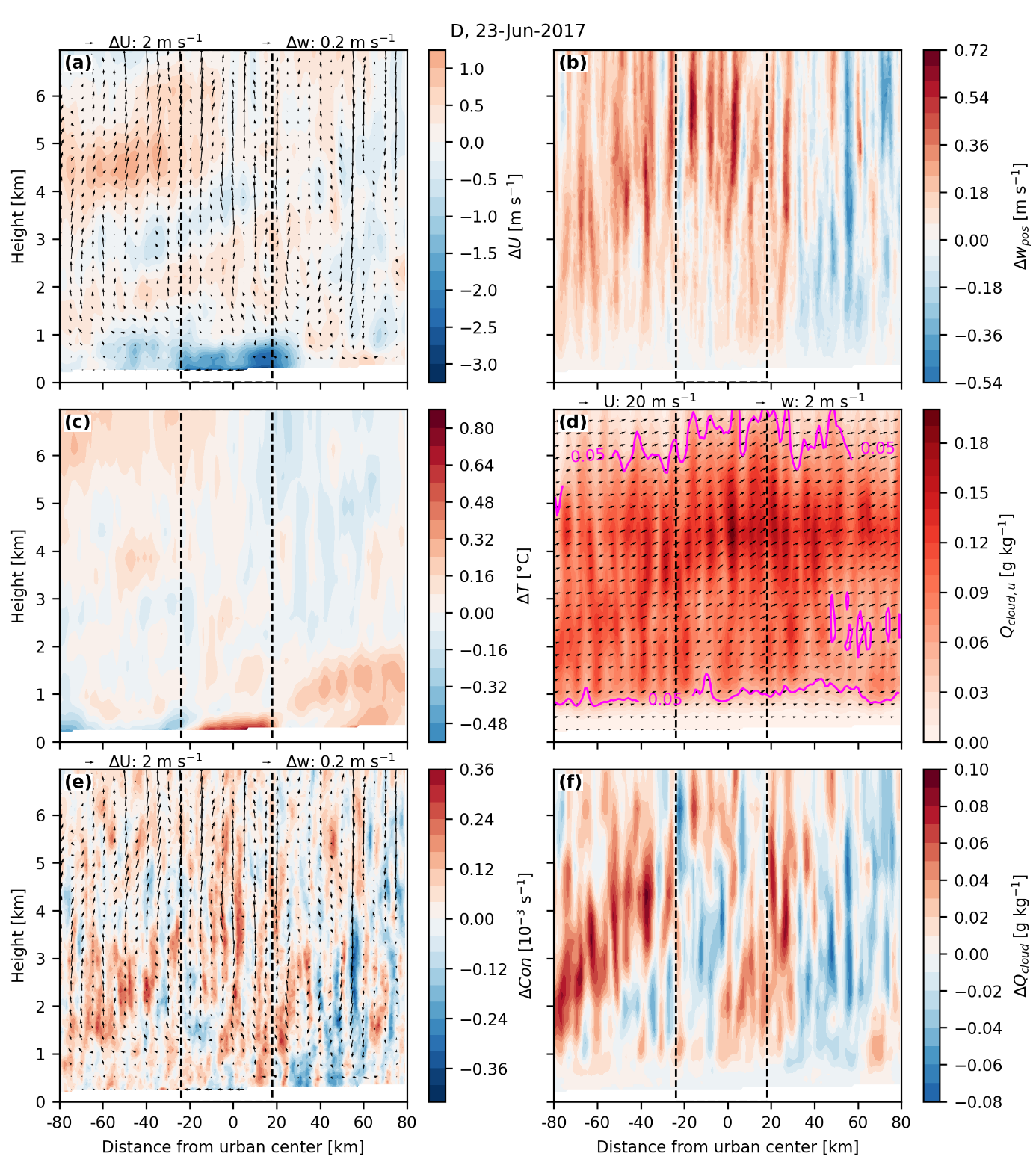
**Figure S10.** As Fig. S8, but for event D.


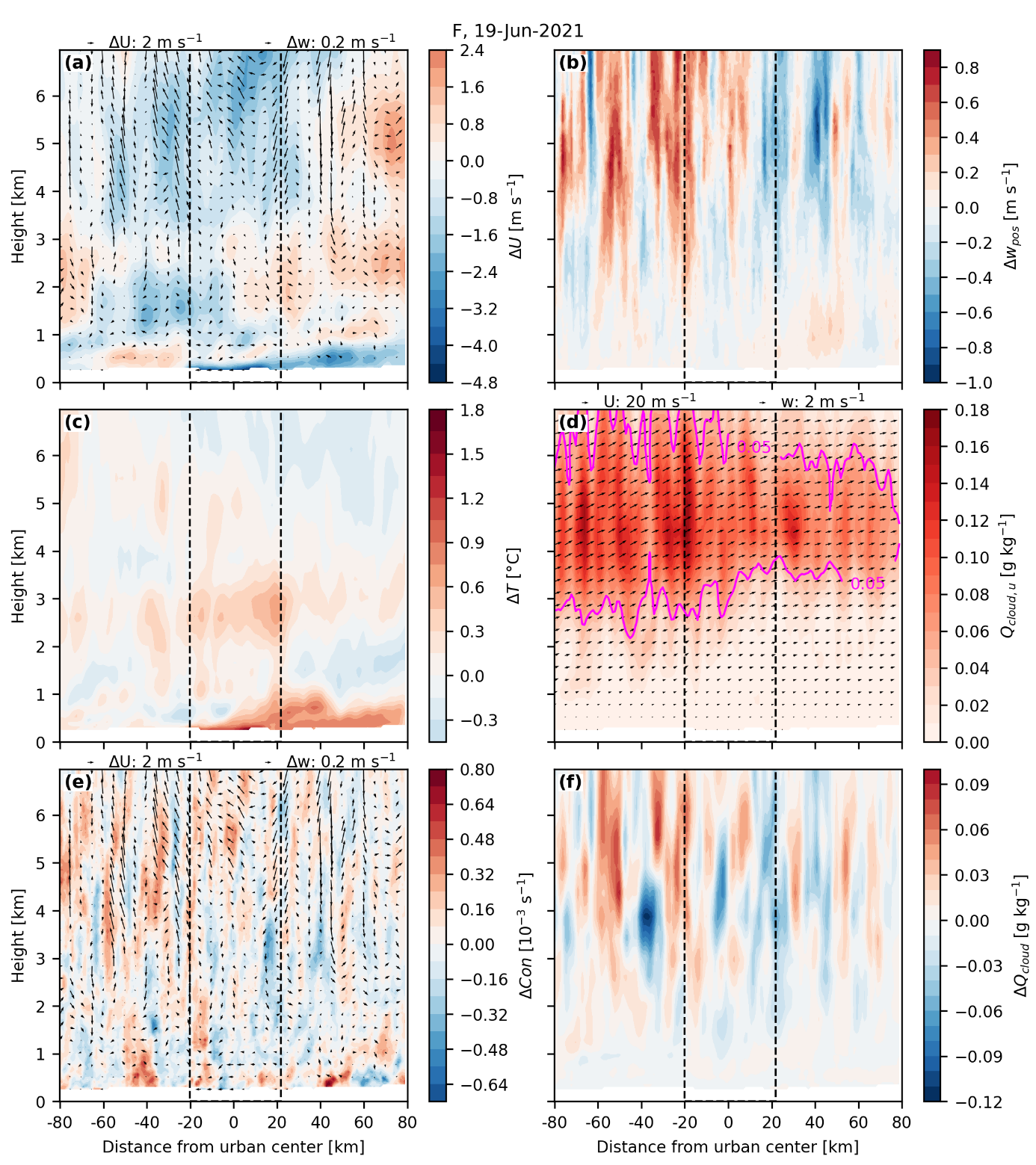
**Figure S11.** As Fig. S8, but for event F.


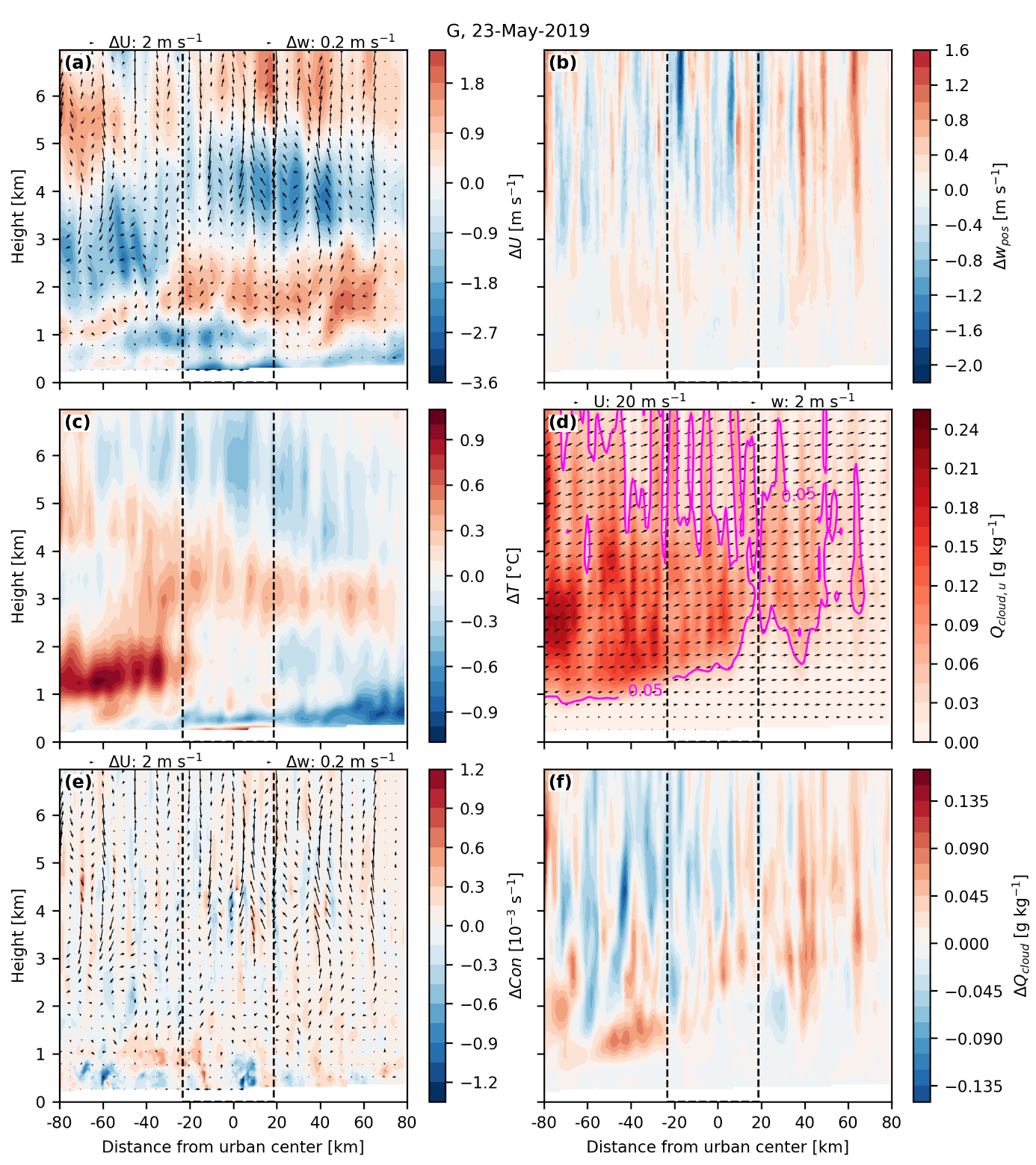
**Figure S12.** As Fig. S8, but for event G.


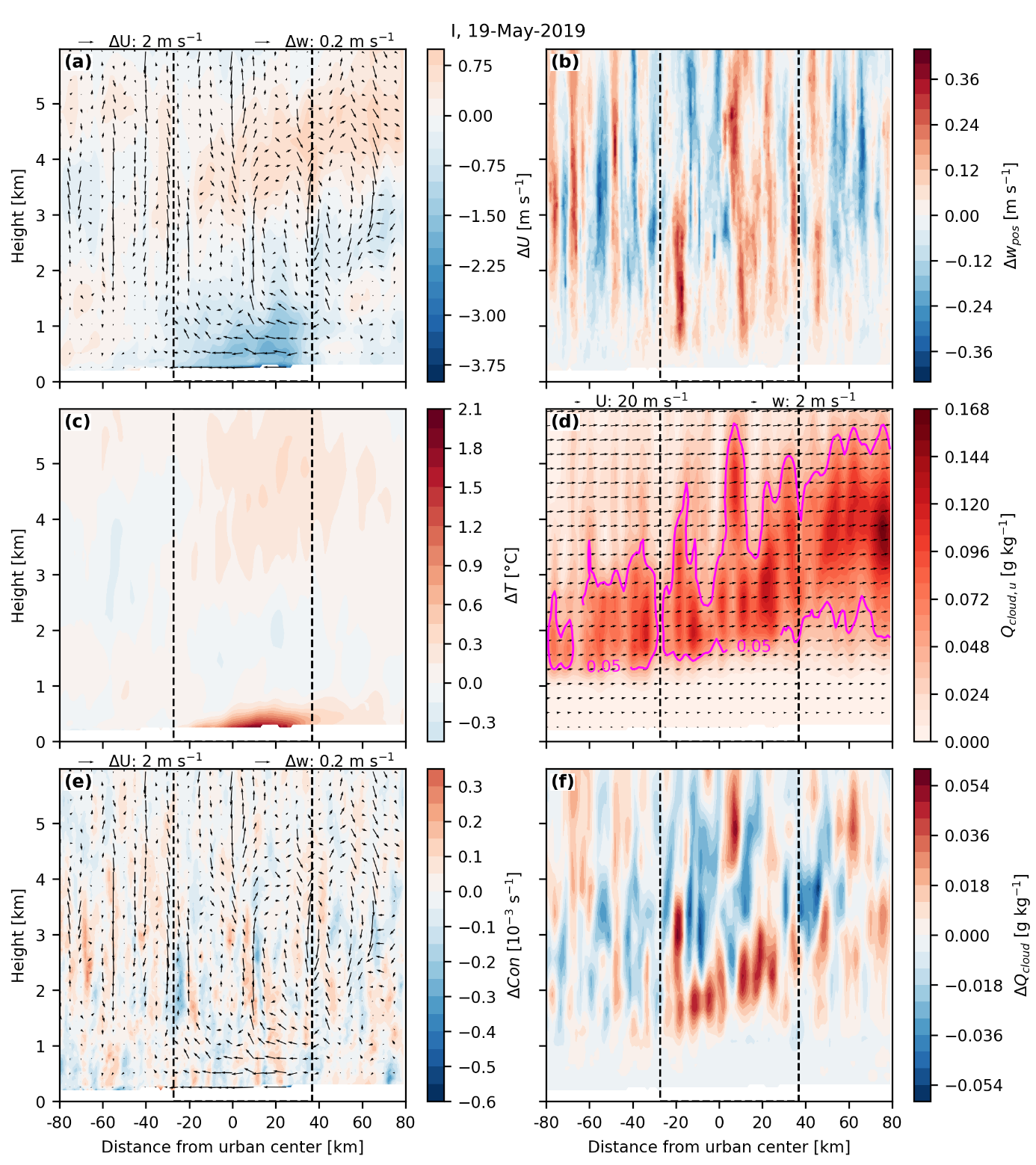
**Figure S13.** As Fig. S8, but for event I.


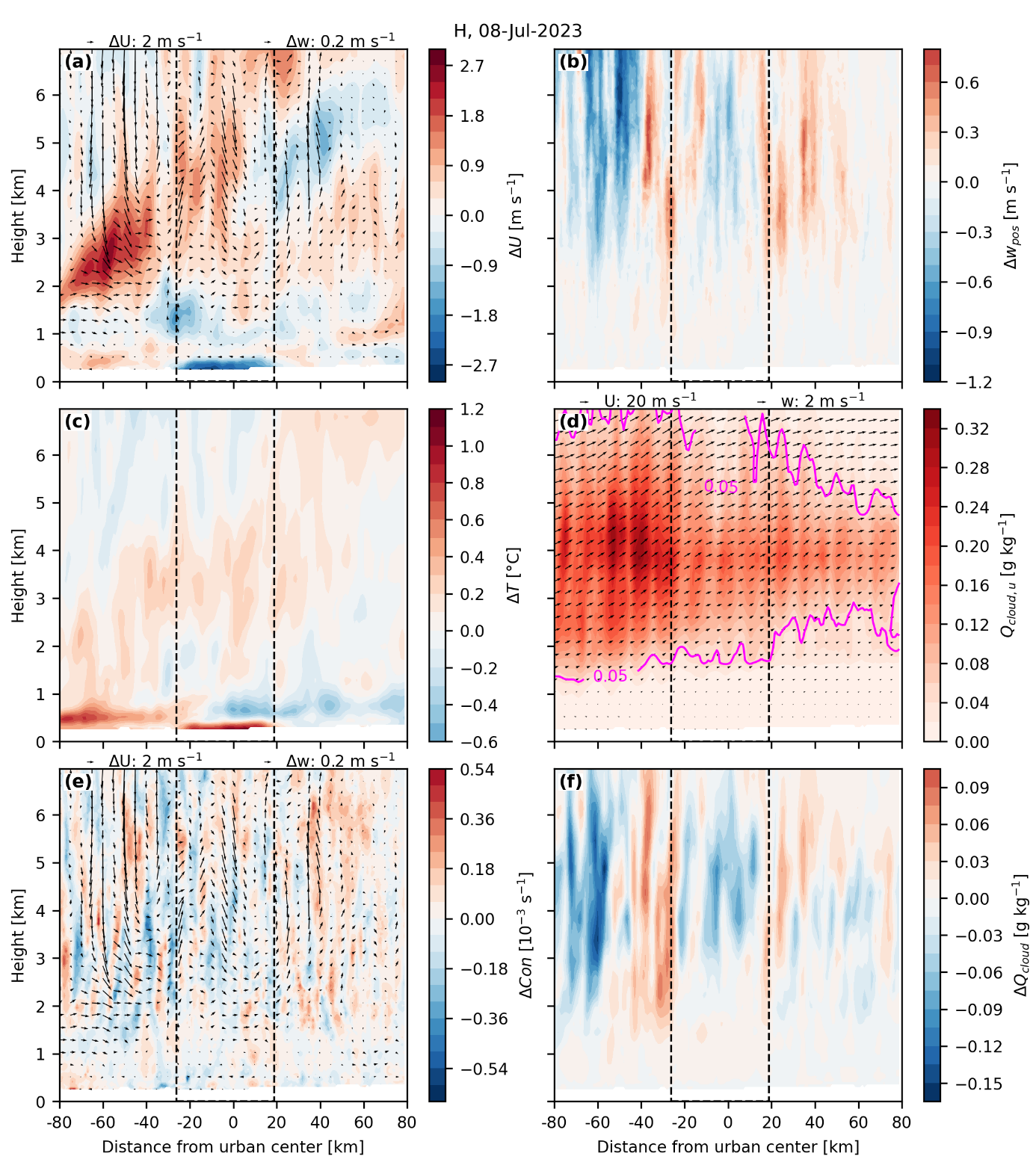
**Figure S14.** As Fig. S8, but for event H.


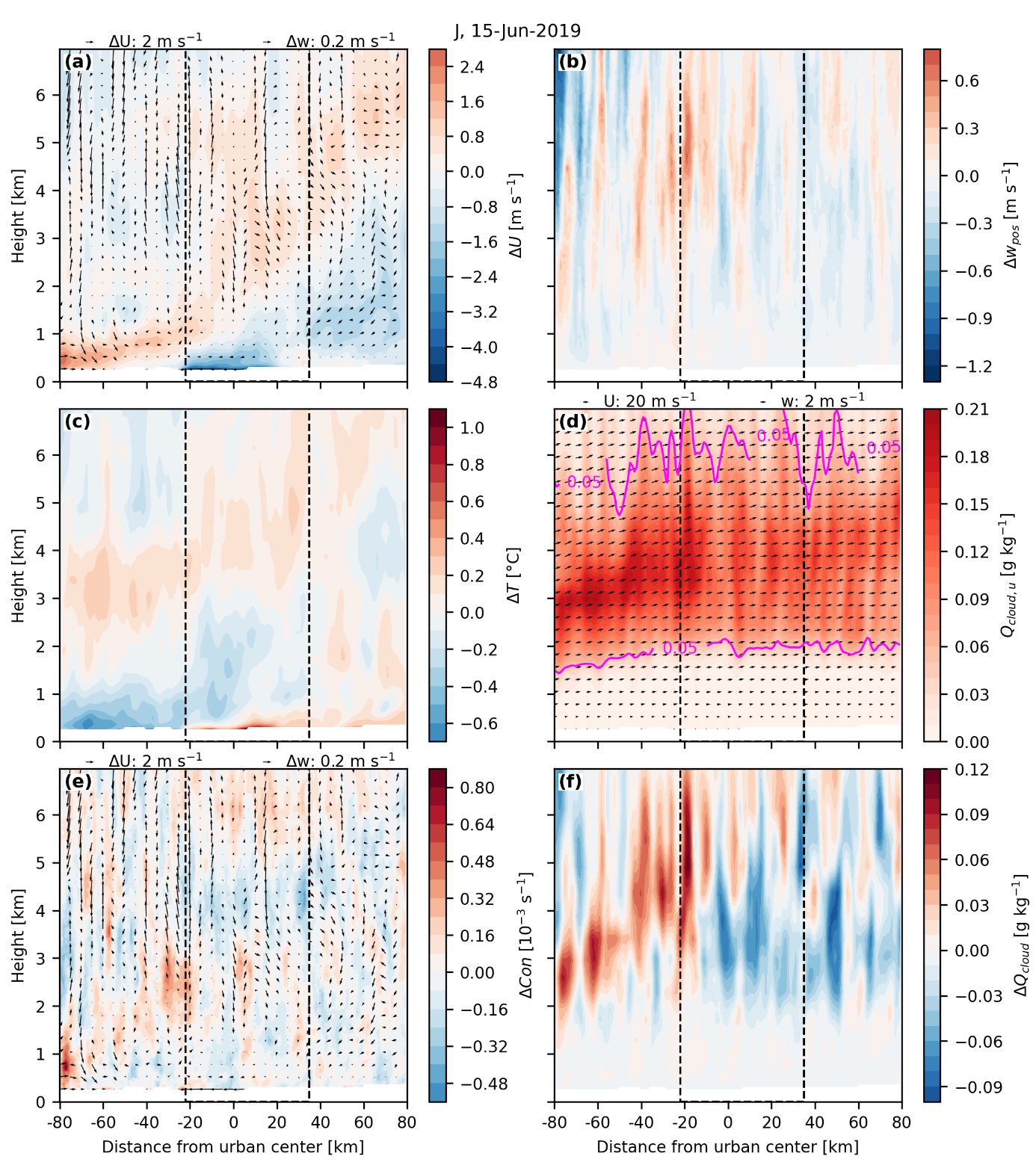
**Figure S15.** As Fig. S8, but for event J.


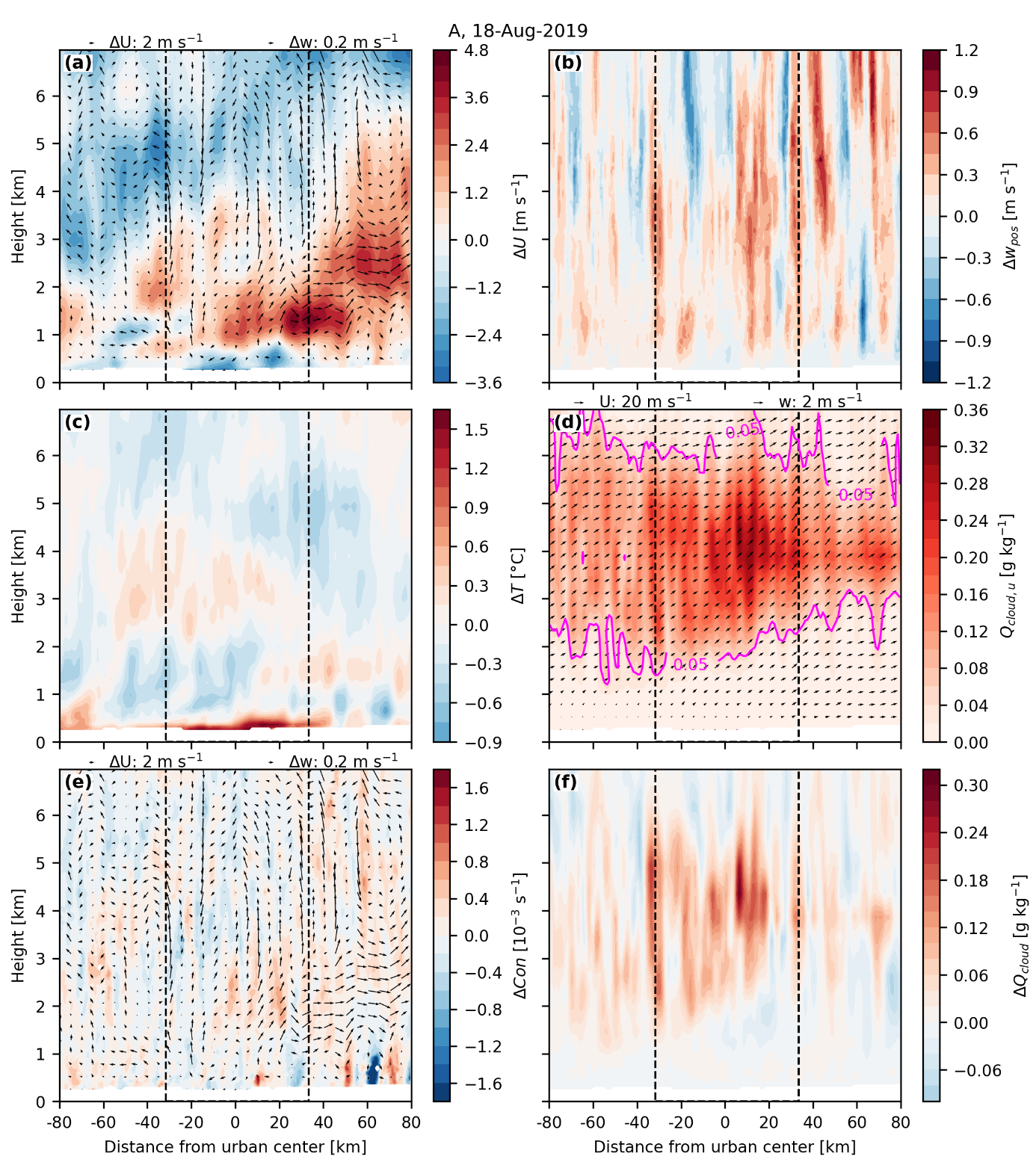
**Figure S16.** As Fig. S8, but for event A.


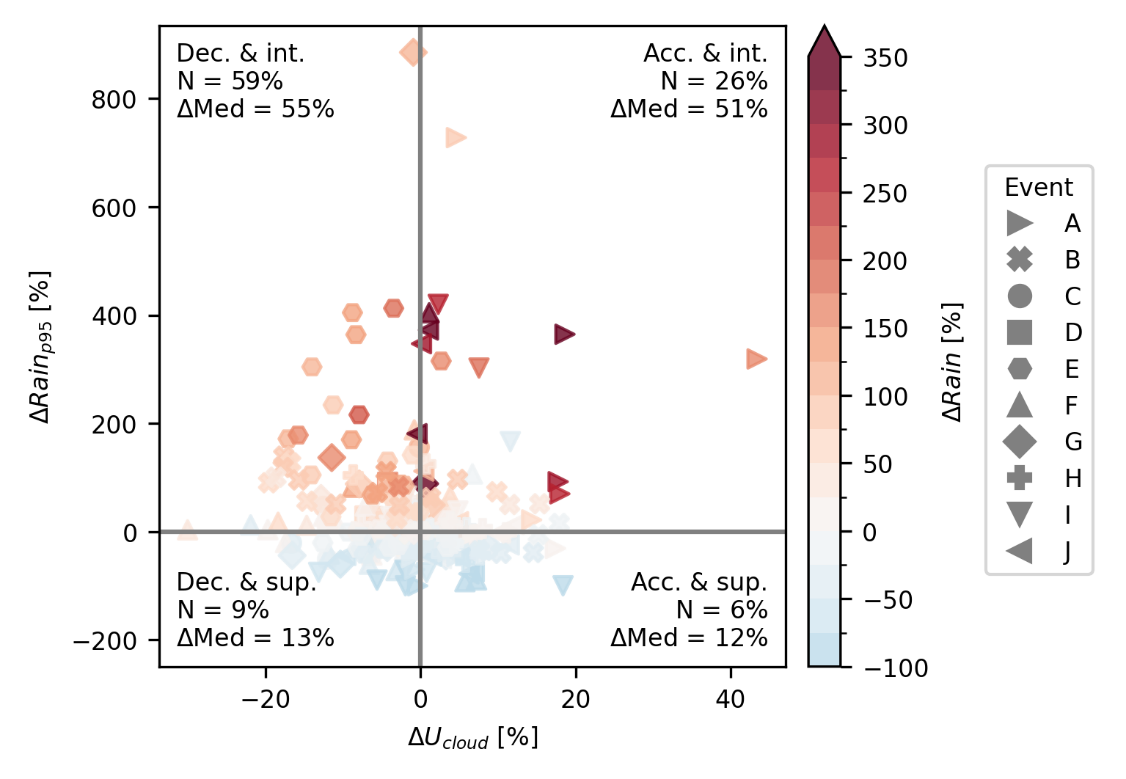


**Figure S17.** Scatter plot of the change in 95^th^ percentile of accumulated rainfall (*∆Rain_p95_*) versus the change in mean cloud-layer horizontal wind speed (*∆U_cloud_*) over the urban area. The color of the points indicates the change in the mean accumulated rainfall (*∆Rain)*. All changes are computed between the ‘urban’ and ‘no-urban’ simulations and computed at each 15-min output time. The shape of the scatter points denotes the rainfall event. ‘Acc’. (‘Dec.’) refers to an increase (decrease) in *U_cloud_* and ‘int.’ (‘sup.’) refers to an increase (decrease) in *Rain_p95_*. *N* and *ΔMed* are computed using only points with positive *ΔRain*; *N* shows the percentage in each quadrant, and *ΔMed* the median *ΔRain* per quadrant.

**
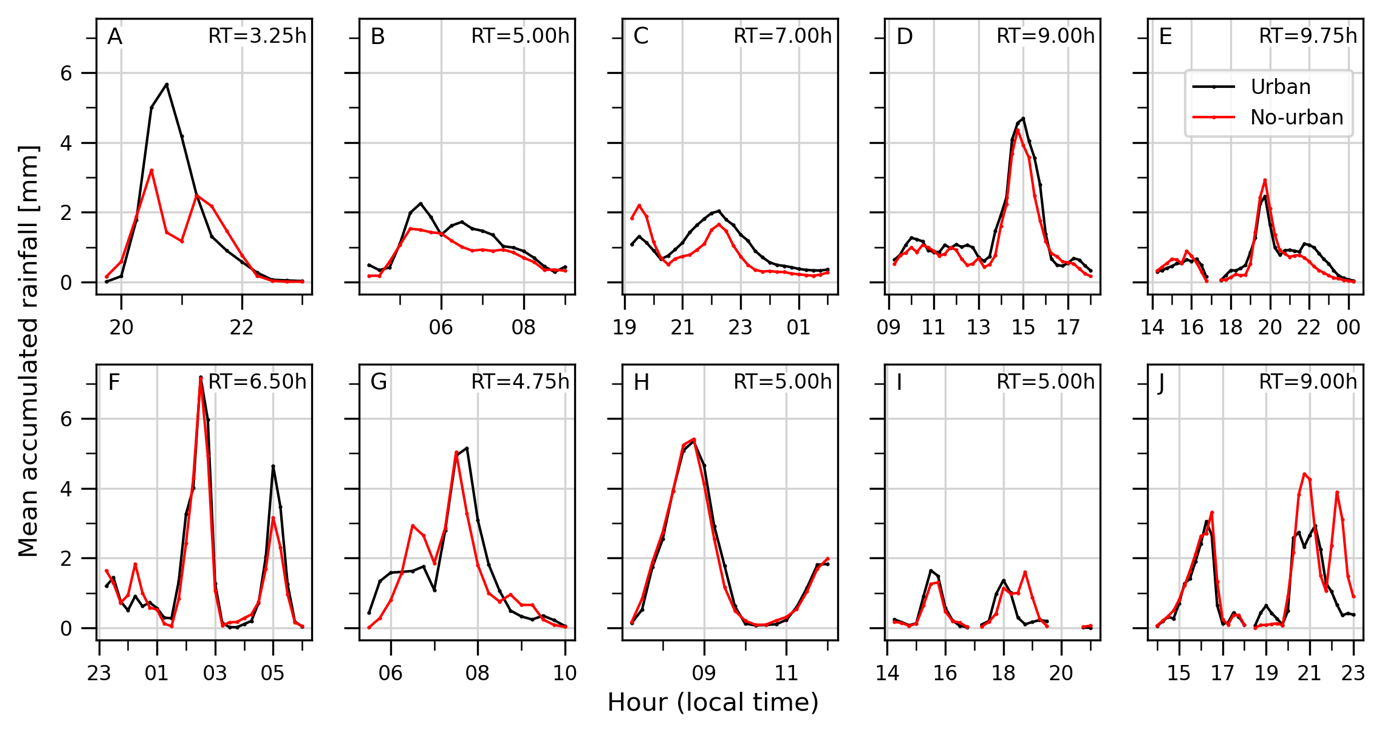
Figure S18.** Time series of 15-min rainfall averaged over the urban area from the ‘urban’ and ‘no-urban’ simulations, in black and red, respectively. Only output times where the mean accumulated rainfall over the urban area was above 0.025 mm per 15 min are displayed. Residence time (RT) is computed as the total period during which the mean accumulated rainfall over the urban area exceeded 0.025 mm, in the ‘urban’ simulation.

**Table S1.** Simulated rainfall event details, including event name, start time (local time), residence time, peak rainfall time (local time), and mean background wind speed at 700 hPa from the ‘urban’ simulation.

| Event | Start time | Residence time [hr] | Peak rainfall time | Background wind [m s^-1^] |
| --- | --- | --- | --- | --- |
| A | 2019-08-18 20:00 | 3.25 | 20:45 | 11 |
| B | 2017-07-11 04:15 | 5 | 05:30 | 15 |
| C | 2015-06-26 19:15 | 7 | 22:15 | 9 |
| D | 2017-06-23 09:15 | 9 | 15:00 | 12 |
| E | 2023-08-09 14:15 | 9.75 | 19:45 | 10 |
| F | 2021-06-18 23:15 | 6.5 | 02:30 | 15 |
| G | 2019-05-23 05:30 | 4.75 | 07:45 | 20 |
| H | 2023-07-08 07:15 | 5 | 08:45 | 11 |
| I | 2019-05-19 14:15 | 5 | 15:30 | 23 |
| J | 2019-06-15 14:00 | 9 | 16:15 | 21 |

References

[1] Torelló-Sentelles, H., Marra, F., Koukoula, M., Villarini, G., Peleg, N.: Intensification and changing spatial extent of heavy rainfall in urban areas. Earth’s Future 12(9), 2024–004505 (2024) <https://doi.org/10.1029/2024EF004505>

[2] Torelló-Sentelles, H., Villarini, G., Koukoula, M., Peleg, N.: Impacts of urban dynamics and thermodynamics on convective rainfall across different urban forms. Urban Climate 62, 102499 (2025) [https://doi.org/10.1016/j.uclim.2025. 102499](https://doi.org/10.1016/j.uclim.2025.%20102499)
